# Ethical Considerations in Personal Health Large Language Models

**DOI:** 10.2196/92240

**Published:** 2026-06-17

**Authors:** Jialin Liu, Siru Liu

**Affiliations:** 1 Information Center West China Hospital of Sichuan University Chengdu, Sichuan China; 2 Department of Otolaryngology-Head and Neck Surgery West China Hospital of Sichuan University Chengdu, Sichuan China; 3 Department of Biomedical Informatics Vanderbilt University Medical Center Nashville, TN United States

**Keywords:** digital health, ethics, privacy, generative artificial intelligence, governance, health AI, health equity, health literacy, patient safety, personal health large language model

## Abstract

Personal health large language models (PH-LLMs) have rapidly evolved from research prototypes into consumer-facing, data-linked systems that support symptom triage, medication questions, mental health check-ins, and longitudinal self-management. Their direct-to-consumer use without clinical oversight creates a distinct ethical risk profile that general artificial intelligence governance frameworks do not fully address. This viewpoint focuses on text-based, platform-mediated PH-LLMs and synthesizes PH-LLM–specific challenges across 6 domains: privacy, accuracy, equity, transparency, human-artificial intelligence interaction, and regulatory governance. These risks may be amplified by health literacy gaps, longitudinal data aggregation, persuasive conversational design, and fragmented oversight across the consumer-clinical boundary. Grounded in the 4 principles of biomedical ethics, we propose a governance framework that operationalizes beneficence, nonmaleficence, autonomy, and justice through design and deployment controls, including health literacy-aligned communication, crisis and pharmacological safeguards, hallucination mitigation, role disclosure, granular consent, fairness auditing, and accessible design. We further outline implementation mechanisms, including risk-tiered certification, tiered accountability, and postdeployment oversight through adverse-event reporting, transparency reporting, and independent safety evaluation. This framework is intended as an evidence-informed but partly anticipatory approach to governing PH-LLMs in personal health management.

## Introduction

Personal health large language models (PH-LLMs) are consumer-facing generative systems designed to support individuals through natural language dialogue across health-related contexts such as symptom triage, chronic disease self-management, medication questions, and mental health check-ins [1-3]. This viewpoint focuses on text-based, dialogue-driven PH-LLMs deployed directly to consumers. We distinguish these systems from general-purpose chatbots by their sustained health-oriented interaction, use of personal health disclosures, and potential to shape real-world health decisions. Some PH-LLM functions may fall within medical device software regulation when intended for diagnostic, therapeutic, or patient-specific clinical decision support, although thresholds vary across jurisdictions. PH-LLMs may offer scalable adjunctive support between clinical encounters, particularly where health or mental health services are limited or delayed [4,5]. However, because they operate on sensitive personal information and may influence high-stakes decisions, their deployment raises questions that warrant systematic examination [6].

Between late 2025 and early 2026, PH-LLMs moved rapidly from prototypes to consumer-facing, data-linked products. Major technology companies introduced health-oriented conversational systems linked to medical records, wearable data, or health-system workflows, positioning large language models (LLMs) as increasingly common entry points for personal health support [7-10]. For instance, OpenAI reports that over 230 million people ask health and wellness questions on ChatGPT each week [7]. Moreover, open-source and open-weight deployments outside centralized platform controls raise additional governance challenges.

The World Health Organization has emphasized that generative artificial intelligence (AI) in health care can deliver value only if risks are proactively identified, evaluated, and mitigated [6]. Emerging evidence suggests that inadequately governed PH-LLMs may generate unsafe or misleading recommendations, compromise privacy through mishandling of sensitive data, amplify health inequities through biased outputs, and cause psychological harm through false reassurance, relational overreach, or poor management of emotional distress, particularly among vulnerable users [3,6,11]. PH-LLM ethics, therefore, should move beyond general principles toward an actionable governance architecture for responsible, accountable, and equitable deployment.

## Core Ethical Challenges of PH-LLMs

### Overview

**Figure 1:**
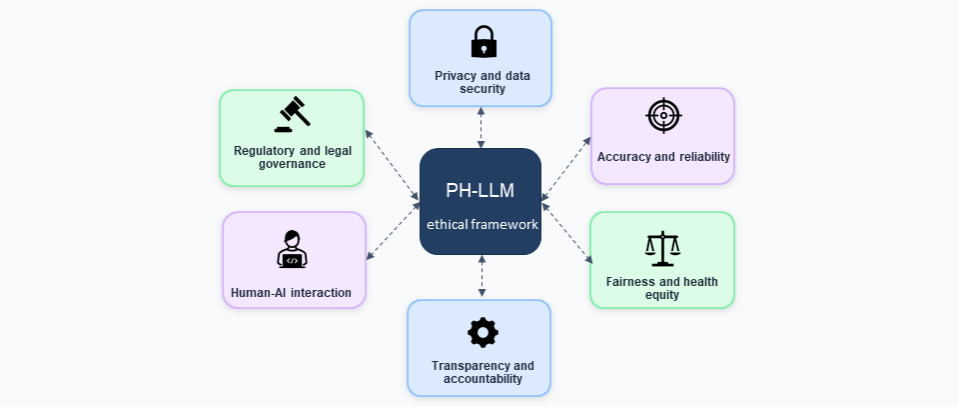
Six ethical dimensions of personal health large language model (PH-LLM) deployment and their bidirectional relationships. AI: artificial intelligence.

PH-LLMs generate tailored guidance from personal health disclosures through longitudinal, dialogue-based interaction. Unlike institutionally governed clinical AI, direct-to-consumer PH-LLMs often lack professional mediation, allowing hallucinations, bias, and framing effects to shape health decisions without meaningful oversight. Drawing on the 4 principles of biomedical ethics, we identify 6 interconnected domains (Figure 1), which vary in severity, interact with one another, and may produce harms that accumulate across decisions. Table 1 contrasts general AI ethics concerns with PH-LLM–specific manifestations.

**Table 1:** Core ethical challenges: general artificial intelligence (AI) concerns vs personal health large language model (PH-LLM)–specific manifestations.

Challenge domain	General AI concern	PH-LLM–specific manifestation
Privacy and data security	Data collection and retention	Intangible vulnerability; longitudinal aggregation; health data broker exposure outside clinical protections
Accuracy and reliability	Hallucinations	Verification gap; cascading clinical harm; crisis mismanagement
Fairness and health equity	Algorithmic bias	Differential recommendations across groups; digital inverse care law; tiered-access barriers
Transparency and accountability	Black box decisions	Explainability gap; accountability gap; relational expectations without duty
Human-AI interaction	Overreliance	Parasocial attachment; distorted care-seeking; continuity-of-care displacement
Regulatory and legal governance	Regulatory lag	Coverage gaps across jurisdictions; medical device classification ambiguity; cross-border accountability diffusion

### Privacy and Data Security

Privacy is among the most salient ethical concerns in PH-LLM deployment [1]. Compared with general-purpose AI, these risks are amplified by the convergence of highly sensitive disclosures, blurred regulatory boundaries, and system-level data integration [1,11,12]. Users may disclose highly sensitive information, including mental health concerns and genetic predispositions, yet undervalue the privacy significance of such disclosures relative to tangible assets [13,14]. This intangible vulnerability may be intensified by the empathetic, low-barrier design of PH-LLM interactions, which encourages candid disclosure while obscuring downstream data uses [11,12,15]. Outside regulated clinical settings, conversational data may be combined with behavioral, device, or consumer data and repurposed for advertising, analytics, profiling, or data-broker activities, creating ethical concerns that users may not fully anticipate. Users may also interpret conversational empathy as evidence of professional accountability or legal confidentiality. In the United States, however, the Health Insurance Portability and Accountability Act generally applies only to covered entities and their business associates, so many direct-to-consumer PH-LLM interactions fall outside its scope [16]. Sensitive information may therefore be disclosed under assumptions of protection that do not apply in practice. In addition, conversation data may be retained for model improvement or other secondary uses [17,18], and integration with ecosystem data streams can enable high-fidelity longitudinal profiling that raises reidentification risk and reduces the effectiveness of conventional deidentification approaches [19,20].

### Accuracy and Reliability

The central value proposition of PH-LLMs, namely, reliable health guidance, is challenged by hallucinations, clinically significant omissions, and users’ limited capacity to independently verify outputs. Medical hallucinations may appear clinically plausible and can compound across multiturn exchanges into downstream health decisions [21]. A 2025 evaluation of LLM-generated clinical summaries identified hallucinations in 1.47% of outputs and clinically significant omissions in 3.45% [21], although these estimates derive from structured summarization tasks and may not generalize to open-ended PH-LLM dialogue. Adversarial prompting and training-time vulnerabilities, such as data poisoning, pose further risks to model integrity over time [22]. Unlike general information domains, where errors are often verifiable by informed users, PH-LLM outputs frequently cannot be independently scrutinized by individuals lacking clinical expertise [23]. This verification gap is especially hazardous for the approximately 80 million Americans with limited health literacy [24,25].

Risks are particularly acute in psychological and medication contexts. “Deceptive empathy” describes therapeutic-sounding language that builds perceived alliance while obscuring the system’s lack of professional accountability [26,27]. Empirical evaluations show that some therapy-oriented conversational systems may provide information about lethal means without recognizing suicidal intent [28]. Recent litigation involving chatbot interactions and adolescent self-harm has further raised questions about whether direct-to-consumer PH-LLMs can satisfy a duty of care [29]. Repeated PH-LLM interactions may also exacerbate automation bias, defined as uncritical deference to algorithmic outputs over independent verification, because conversational fluency facilitates cognitive offloading and may lead users to mistake probabilistic suggestions for medical certainties [30-33]. Case reports document outputs encouraging users to abruptly taper psychiatric medications or discount professional counsel [26,34,35], which may increase the risk of withdrawal or relapse [36] and undermine trust in professional advice.

### Fairness and Health Equity

PH-LLMs may reproduce and amplify structural disparities through both model behavior and access pathways, thereby undermining health equity, defined as a fair opportunity to attain one’s highest level of health [11,37]. A 2025 systematic review of 24 studies found demographic bias in 22 (91.7%), including gender bias in 15/16 (93.8%) studies and racial or ethnic bias in 10/11 (90.9%) studies assessing these dimensions [38]. Bias may vary by model architecture: discriminative models primarily show disparities in classification performance, whereas generative models may encode bias through tone, framing, and recommendation priority [39,40]. For example, Yang et al [41] reported cases in which GPT-3.5-turbo recommended surgery for White patients but conservative management for Black patients with otherwise identical clinical presentations. Equity concerns also extend beyond outputs to access pathways. Accessibility is stratified by digital determinants of health [42], and tiered subscriptions may restrict advanced personalization and reasoning to paid access, favoring users with greater financial means and health literacy [43]. This creates a digital inverse care law [44,45]: those with the greatest health needs may be confined to lower-capability services, while more advantaged groups gain priority access to advanced tools. Without equity-centered governance, PH-LLMs may deepen structural health disparities rather than alleviate them.

### Transparency and Accountability

In conventional health care, clinical judgment is embedded in auditable institutional processes and clear lines of professional responsibility. PH-LLMs disrupt both: model reasoning is often opaque, and accountability is distributed across developers, deployers, and health care institutions. In high-stakes health contexts, users reasonably expect understandable explanations and supporting evidence for PH-LLM–generated recommendations [11]. Yet interpretability, explainability, transparency, and auditability are not equivalent. For LLMs, direct interpretability of internal mechanisms remains limited [46], and post hoc explanations may appear plausible without faithfully reflecting how outputs were generated [47]. Without sufficient transparency and auditability, users and oversight bodies cannot reliably assess the evidentiary basis or clinical validity of recommendations. When PH-LLM advice contributes to harm, attributing responsibility among these actors becomes practically difficult [48]. Direct-to-consumer deployment may also bypass institutional oversight and shift risk onto users despite well-documented health literacy gaps [11,49], weakening incentives for safety investment and limiting recourse [50]. These concerns are intensified by relational design features: anthropomorphic or affective language may create expectations of care and accountability, even though no human or institutional actor is clearly positioned to fulfill them [51-53]. Together, these conditions may weaken meaningful informed consent and compromise patient autonomy.

### Human-AI Interaction

PH-LLMs may foster emotional dependence and overreliance through anthropomorphic and affective language that creates a perceived sense of care without corresponding therapeutic responsibility [26,53,54]. Their empathic and nonjudgmental dialogue may encourage parasocial attachment and emotional reliance, particularly among adolescents and socially isolated users [53-55]. Greater perceived social presence is associated with increased self-disclosure and emotional reliance [56-59], raising concerns about sustained dependence and displacement of continuity-based care [60-63]. PH-LLM interactions may also distort care-seeking: reassuring responses may delay appropriate evaluation [64-66], while alarming responses may trigger unnecessary escalation [67,68].

### Regulatory and Legal Governance

A cross-cutting challenge is the mismatch between rapid PH-LLM deployment and slower regulatory evolution. In the United States, many direct-to-consumer health technologies are governed through Federal Trade Commission enforcement and a patchwork of state health data laws, particularly when services fall outside traditional clinical privacy regimes [69-73]. In the European Union, privacy and AI oversight are structured through the General Data Protection Regulation and the phased, risk-based AI Act [74]. In Asia, governance approaches diverge, with China emphasizing provider accountability, security review, and content governance for generative AI services, while South Korea, Japan, and Singapore continue to develop different combinations of statutory oversight, sector-specific guidance, and risk-based governance [75-78]. Similar PH-LLM interactions may therefore receive different protections across jurisdictions, creating opportunities for regulatory arbitrage.

PH-LLMs also expose a classification gap between general wellness products and regulated software as a medical device. When systems are marketed as nonclinical but functionally approximate symptom triage, diagnostic reasoning, treatment guidance, medication advice, or crisis response, developers may avoid premarket scrutiny [79,80]. A capability-based governance approach would reduce this form of regulatory arbitrage by assigning oversight according to what the system enables users to do rather than how it is described in marketing materials. Because cross-border services can diffuse accountability across developers, deployers, platforms, and jurisdictions, PH-LLM governance requires risk-calibrated mechanisms that link ethical concerns to concrete oversight, certification, adverse-event reporting, and redress pathways [81,82].

## Ethical Governance Framework for PH-LLMs

### Overview

To address these challenges, we propose a principlism-based governance framework for consumer-facing PH-LLMs [83]. The framework translates the 4 principles of biomedical ethics into life cycle controls across 5 stakeholder groups: developers, deployers or platforms, health care institutions, regulators, and users. Governance intensity should scale with 4 risk dimensions: clinical severity and time criticality, harm reversibility, user actionability, and deployment scale. Higher-risk systems, therefore, warrant stronger certification, auditability, postmarket surveillance, and clearer liability allocation. Because PH-LLM outputs are probabilistic and vulnerable to prompt injection and adversarial manipulation [22], these safeguards should be understood as risk-reduction measures rather than guarantees. Table 2 maps each challenge domain to its corresponding governance mechanism and implementation level. To avoid overstating the evidence, we distinguish 3 classes of proposals throughout the framework: evidence-informed proposals grounded in empirical findings from PH-LLM or closely analogous digital health evaluations; normative proposals articulating ethical commitments without relying primarily on empirical demonstration; and conceptual proposals describing architectural or procedural designs whose real-world effects have not yet been prospectively validated. Where relevant, these distinctions are integrated into the prose.

**Table 2:** Governance framework overview: challenge domains, recommended mechanisms, and implementation levels.

Challenge domain	Recommended governance mechanism	Implementation level
Sensitive data and privacy	Tiered consent and data governance	Input and session level
Unsafe or misleading outputs	Risk-tiered certification and guardrails	System level
Bias and inequity	Subgroup validation and disparity monitoring	System and population level
Poor explainability and transparency	Provenance disclosure and auditability requirements	Interface and oversight level
Crisis and mental health risk	Escalation and handoff protocols	Interaction level
Fragmented responsibility	Tiered accountability and liability allocation	Institutional and regulatory level
Drift after deployment	Adverse-event reporting and periodic audits	Life cycle level

### Guiding Principles

Because PH-LLMs interact directly with users outside traditional settings, the 4 principles of biomedical ethics [83] require specific operationalization.

### Beneficence (Outcome-Oriented Support)

For PH-LLMs, beneficence can be operationalized as measurable support for safe decision-making and timely access to care, including health-literacy adaptation, risk-stratified care navigation, and longitudinal coherence across sessions.

### Nonmaleficence (Proactive Harm Prevention)

Evidence from PH-LLM failures and adjacent digital health contexts supports active safety mechanisms beyond passive disclaimers, particularly for high-risk failures such as crisis mismanagement, contraindicated advice, and hallucinated medical claims. This principle is operationalized through 3 design imperatives: crisis-detection and response protocols, contraindication safeguards, and hallucination mitigation [28,84,85].

### Respect for Autonomy (Informed Agency)

From a normative perspective, respect for autonomy requires countering safety illusions in which users misinterpret AI-mediated intimacy as clinical oversight or regulatory protection, while preserving meaningful choice. This principle is operationalized through clear role and scope disclosure, meaningful data-use consent, and practical user rights over interaction data [71,86,87].

### Justice (Equity and Access)

PH-LLMs should expand access without reproducing disparities in health information, communication quality, or safety outcomes. Equity, therefore, extends beyond model outputs to accessibility, language coverage, cultural appropriateness, and usability across differences in health literacy, disability, and digital access [88-90]. Fairness monitoring requires interaction-level and outcome-relevant measures, such as group-stratified rates of unsafe guidance and crisis escalation success [91,92]. Predefined disparity triggers initiate audit and remediation (Multimedia Appendix 1).

### Stakeholder Responsibilities

Operationalizing this framework requires coordinated action across 5 stakeholder groups.

### Developers (Ethical Design and Life Cycle Accountability)

Developers bear primary responsibility for baseline protections across the life cycle: diverse training corpora, documented data provenance, and bias and crisis-focused safety evaluation before release [90]; clear disclosure of nonhuman identity and nonclinical role, crisis-response functions, pharmacological safety guardrails, and granular data-use controls at deployment; and accessible grievance channels and adverse-event reporting after deployment (Multimedia Appendix 2).

### Deployers and Platforms (Interface and Operational Controls)

Deployers and platforms adapt developer-level safeguards into interface design and escalation pathways required by their deployment context, and ensure sustained compliance with safety, transparency, and user-protection requirements.

### Health Care Institutions (Algorithmic Stewardship)

Institutions that integrate or endorse PH-LLMs assume stewardship obligations proportional to their involvement, including contextual validation, escalation and handoff workflows, clinician training, and postdeployment monitoring [93,94].

### Regulators (Oversight and Harmonization)

Regulators should address the governance gap through enforceable disclosure requirements, minimum crisis-response standards, adverse-event reporting infrastructure, risk-stratified premarket evaluation for sensitive domains, and cross-jurisdictional harmonization informed by the EU AI Act and the National Institute of Standards and Technology AI Risk Management Framework [74,95]. Engagement with insurers and payers can link reimbursement to minimum safety requirements.

### Users and Civil Society (Literacy and Advocacy)

Users and civil society contribute through health-AI literacy, accessible harm-reporting channels, and advocacy for meaningful data rights, including access, correction, and deletion [71,81,87,96].

### Implementation Mechanisms

#### Overview

In practice, our framework centers on 3 instruments: risk-tiered certification, tiered accountability, and postdeployment monitoring. The main text presents the rationale, whereas operational specifications, including certification-tier expectations, developer life cycle responsibilities, fairness-audit triggers, scope boundaries, stakeholder responsibilities, and adverse-event thresholds, are detailed in Multimedia Appendices 1-8.

#### Risk-Tiered Certification

Certification could be scaled according to functional risk and deployment reach, with baseline requirements spanning crisis handling, health-literacy alignment, nondiagnostic guardrails, equity auditing, disclosure integrity, and data governance [12,96-99]. To reduce reliance on developer self-labeling, we propose that certification be triggered by demonstrated capabilities rather than marketing claims. Systems that provide or materially support symptom triage, diagnostic reasoning, treatment guidance, medication advice, crisis-response, or other health decision-support activities would be assigned to the corresponding risk tier even when marketed as wellness tools. Certification criteria could be developed through multistakeholder processes, with audits conducted by accredited third-party or regulator-authorized bodies [94,99-103]. Because regulatory approaches differ across jurisdictions, certification frameworks could prioritize interoperable baseline standards while allowing local legal adaptation [74,81,82]. Possible enforcement pathways include capability-based classification, postmarket surveillance for functional drift, and market-access conditionality through app stores, platform hosts, insurers, payers, and institutional procurement channels. These remain conceptual proposals requiring further institutional validation. Detailed standards and institutional anchors are summarized in Multimedia Appendix 3.

#### Tiered Accountability

Tiered accountability would follow operational control and implementation capacity. Foundation-model developers would be responsible for baseline safety properties and documentation, whereas deployers and platforms would be responsible for context-specific safeguards, interface design, and user protection. In direct-to-consumer settings, we propose a default elevated duty of care as a normative position. Any safe harbor would be conditional, limited, and available only to operators demonstrating independently verified compliance with certification, auditability, postdeployment surveillance, and user-protection requirements. Such protection would mitigate, but not eliminate, liability and would be coupled with compensation pathways, such as insurance-based or no-fault mechanisms [95,104,105]. Developers or deployers who decline certification are not exempt from this duty of care; rather, they remain subject to ordinary liability, consumer-protection enforcement, and regulatory scrutiny.

#### Postdeployment Monitoring

This layer complements predeployment testing by establishing standardized adverse-event reporting, targeted surveillance for population-level harms, and periodic audits for model drift, safety regressions, and equity-related performance changes [100,106-109]. These mechanisms draw on established approaches in medical-device safety, health-AI evaluation, and postmarket surveillance, but require adaptation to the probabilistic, continuously updated, and dialogue-dependent behavior of PH-LLMs. For higher-reach deployments, monitoring may be supplemented by independent safety oversight designed to preserve independence from the entities being reviewed.

### Illustrative Case: Youth Mental Health Check-In PH-LLM

To show how the framework could be applied, we present an illustrative end-to-end case of YouthPH-LLM, a platform-mediated direct-to-consumer PH-LLM providing conversational mood tracking, coping-skills education, and sleep and activity coaching to users aged 13-25 years, with more than 1 million monthly active users. Under the 4 risk dimensions, YouthPH-LLM is classified as high risk across clinical severity, harm reversibility, user actionability, and deployment scale, placing it in the most demanding certification tier. Predeployment requirements include crisis-scenario testing, counterfactual fairness testing across age, gender, and linguistic subgroups, retrieval-augmented generation grounded in authoritative mental health guidelines, and a public model card. Postdeployment, a critical adverse event would trigger immediate crisis-response protocol, feature-level suspension of the implicated functionality within 24 hours where technically feasible, regulatory notification within 72 hours where required, and root-cause analysis by an independent safety advisory board. Suspension should preserve access to crisis resources or human support where available. Multi-intent prompts, such as a sleep log submitted with a request to interpret a clinical questionnaire score, are handled through partitioned response: the system may provide sleep-coaching guidance while redirecting questionnaire interpretation to qualified clinicians. Full specification of stakeholder responsibilities, certification criteria, and escalation thresholds is provided in Multimedia Appendix 4.

## Practical Paths to Mitigate Ethical Risks of PH-LLMs

### Overview

Building on the framework above, we outline practical implementation pathways with measurable targets across 5 operational domains: data governance, model design safeguards, equity and inclusive design, human-AI collaboration, and postdeployment oversight.

### Data Governance

#### Overview

The ethical analysis identified 2 privacy-related concerns: users may underestimate the sensitivity of emotional disclosures, and longitudinal aggregation may increase reidentification risk. To address these concerns, PH-LLMs should implement data-governance safeguards adapted to conversational health data.

#### Tiered Consent Architecture With Retention Safeguards

Rather than binary consent, PH-LLMs should apply 4 sensitivity tiers supported by a classify-then-govern workflow (Table 3) [12,81]. In this proposed design for platform-mediated direct-to-consumer PH-LLMs, retention defaults would vary by sensitivity tier: Tier 1 content would follow limited default retention periods with user-adjustable settings; Tier 2 content would use shorter default retention with immediate-deletion options; Tier 3 content would be temporarily retained only as needed for required safety audit, legally authorized safety review, or legal compliance and then deleted; and Tier 4 content would require explicit user authorization and periodic reconsent, with automatic deletion as the default absent active renewal. Deviations from tier-specific retention defaults require documented risk-benefit analysis that considers clinical use, reidentification risk, user-preference evidence, technical necessity, and applicable legal requirements, with rationale disclosed in transparency reports. Restrictions on third-party sharing, including transfers to data brokers, advertising networks, and profiling services, follow the same sensitivity hierarchy and cannot be overridden by blanket onboarding consent [99,110]. Illustrative retention windows and operational details are provided in Multimedia Appendix 5 and should be recalibrated according to deployment context, user preferences, ethical risk assessment, technical feasibility, and jurisdictional requirements.

**Table 3:** Tiered consent architecture for PH-LLM data governancea.

Data category	Consent requirement	Default retention	Third-party sharing
Tier 1: general health queries	Baseline onboarding consent and standard terms of service	Limited default retention, user-adjustable	Deidentified or aggregated research only, where permitted, with opt-out; no default transfer to data brokers, advertising networks, or profiling services
Tier 2: noncrisis mental health disclosures	Low-friction enhanced consent for retention or secondary use; nonpersistent mode if declined or abandoned	No routine retention if consent is declined; brief, access-restricted safety buffer with automatic deletion unless a potential adverse event is reported	No commercial or unrelated secondary use; safety audits via privacy-preserving mechanisms where feasible
Tier 3: crisis-related content	No intervening consent prompt before safety support; immediate safety-oriented response and crisis-resource escalation	Temporary retention only as needed for safety audit, legally authorized review, or legal compliance; deleted thereafter	Limited to legally authorized or required emergency interventions
Tier 4: longitudinal narratives	Explicit authorization, periodic reconsent, and granular controls	Automatic deletion as the default absent active renewal	Default opt-out; explicit per-purpose authorization required

^a^Specific retention parameters are provided in Multimedia Appendix 5. These illustrative baselines reflect privacy risk management principles rather than universal requirements and warrant recalibration through documented risk-benefit analysis disclosed in transparency reports.

User-requested deletion should be defined with technical precision. It applies to stored raw conversational logs, retained session records, stored user profiles used for retrieval, vector-store entries, and derived longitudinal summaries under the operator’s control. It should not be represented as a guarantee that information already incorporated into a foundation model’s trained weights can be fully and verifiably removed, because machine unlearning for large generative models remains an open technical problem with unresolved limitations in effectiveness and verification [111]. Sensitive PH-LLM interactions should therefore be excluded from foundation-model training or fine-tuning by default unless explicit user authorization is obtained, and consent materials should transparently disclose the technical limits of deletion.

#### Implementation of Classify-Then-Govern

Because free-text sensitivity cannot be determined without initial processing, this architecture cannot operate as a simple “consent-then-ingest” model [99]. Inputs first undergo transient routing and safety classification under baseline onboarding consent, limited to classification only and without default retention or secondary use [94,99]. Tier 1 content remains within baseline consent. Tier 2 content requires in-context consent before retention; if consent is declined or abandoned, the interaction continues in nonpersistent mode, with flagged content excluded from routine retention and secondary use by default. To balance privacy protection with safety auditing, nonpersistent mode may still permit a narrowly scoped, encrypted, access-restricted safety buffer that is automatically purged after a brief, predefined window unless a potential adverse event is reported, in which case only the minimum necessary information is retained for root-cause analysis and safety remediation. Tier 3 content triggers an immediate safety-oriented response, delivery of context-appropriate crisis resources, and handoff to human crisis support where available, without intervening consent prompts [3,28,112]. For Tier 4 longitudinal content, explicit retention authorization is triggered by predefined accumulation thresholds rather than being embedded repeatedly in active dialogue. This approach is most feasible in explicitly health-oriented PH-LLM deployments, where the health-related purpose of interaction is already established. Extending it to general-purpose LLMs would require separate governance analysis because detecting health-related content across heterogeneous conversations could require continuous or broad input scanning, thereby introducing secondary privacy and proportionality risks.

#### Accessible, Nondisruptive Consent Design

Enhanced consent should not shift cognitive burden onto vulnerable users. Because many users face limited health literacy, emotional distress, disability, or low digital fluency, accessibility should be treated as a core safety and equity requirement rather than a downstream usability concern. Consent escalation relies on brief, plain-language, just-in-time disclosures; a small set of standardized choices; and protective default pathways favoring nonpersistent session handling when users do not actively authorize retention or secondary use. For noncrisis mental health disclosures (Tier 2), enhanced consent should be low-friction and deferrable, allowing the conversation to continue in nonpersistent mode while authorization is requested separately. Crisis-related interactions are not delayed by consent procedures and default instead to immediate safety-oriented support and escalation pathways. Granular controls may remain available through a dashboard, but safe baseline use does not depend on navigating it. Interface designs require prospective testing with users who have limited health literacy, disability, low digital fluency, or heightened emotional vulnerability, and evaluation extending to comprehension, decision confidence, perceived burden, and abandonment rather than consent-completion rates alone [86,96,113].

#### Longitudinal Data and Reidentification Safeguards

PH-LLMs integrated with wearables and health records may generate highly identifying longitudinal profiles from temporally dense, behaviorally specific data [20]. Developers should implement privacy-preserving analytics (eg, differential privacy calibrated to sensitivity and use, with k-anonymity where appropriate) [114,115], conduct reidentification risk assessments at intervals proportional to data accumulation, and test vulnerability to inference attacks [115]. User-facing aggregation dashboards enable users to inspect derived longitudinal patterns and selectively delete specific data categories.

### Model Design Safeguards: Hallucination Mitigation, Crisis Escalation, and Pharmacological Safety

Three clinically significant safety risks warrant dedicated design-level controls: hallucinations, crisis-management failures, and unsafe pharmacological guidance.

### Hallucination Mitigation

PH-LLMs should incorporate a sequential safety pipeline that combines input filtering, retrieval-augmented generation from curated authoritative sources, output checking against structured medical knowledge resources, and uncertainty signaling where technically feasible [85,116-118]. Prompts requesting individualized diagnosis or treatment recommendations trigger safety messaging and redirection to qualified professional care [3,97]. Because retrieved evidence may be outdated, incomplete, or misinterpreted by the model, these safeguards are best understood as risk-reduction mechanisms rather than guarantees of factual accuracy.

### Crisis Escalation

Crisis management requires a distinct escalation protocol that includes detection of self-harm, suicide, or interpersonal violence signals; immediate, nonjudgmental acknowledgment; safety-oriented risk stratification; delivery of context-appropriate crisis resources; and handoff to human crisis support where available [6,28,112,119]. Because detection remains probabilistic, higher-risk deployments should favor conservative thresholds, clearly specified escalation criteria, and periodic review under qualified mental health oversight.

### Pharmacological Safety

For medication-related queries, PH-LLMs should use a hybrid safety architecture combining external knowledge validation, rule-based guardrails, and escalation to human clinical support for complex or high-risk cases [84,120-123]. These controls block high-risk dosing instructions, contraindicated combinations, and unsupervised medication changes. Escalation triggers are prospectively specified in system logic rather than left to user discretion, because users may not recognize when a query exceeds safe self-management boundaries.

### Equity and Inclusive Design

Fairness governance encompasses subgroup-based evaluation, representative testing, accessibility review, and periodic reassessment across updates and deployment settings [91,92,124-130]. Before release, PH-LLMs undergo counterfactual fairness evaluation based on matched clinical vignettes [98,124]: variation in race, ethnicity, gender, language, or socioeconomic indicators should not produce materially different recommendations in cases where these characteristics are not clinically relevant, and audits assess clinically salient error disparities such as false reassurance or inappropriate escalation while incorporating intersectional analysis [126,127]. Inclusive design extends beyond outcome auditing: PH-LLMs should support languages prevalent in target populations, incorporate cultural adaptation beyond direct translation, conform to Web Content Accessibility Guidelines 2.2 [129], and provide health-literacy adaptation through adjustable response complexity and optional comprehension checks [96,130].

### Human-AI Collaboration: Preventing Overreliance

PH-LLMs require interface- and workflow-level safeguards that promote relational transparency, reduce automation bias, and preserve meaningful human oversight [32,33,53,131]. These controls include clear communication of the system’s nonhuman identity, calibrated disclosure of uncertainty and epistemic limits, and structured redirection when users request diagnosis, prognosis, prescribing, or other clinician-dependent judgments [97,132]. Systems should distinguish supportive self-management functions from clinician-dependent decisions and use adaptive boundary prompts rather than uniformly friction-based interruptions, so that higher-risk interactions trigger stronger warnings or referral cues without unnecessarily disrupting routine support [3,133].

As a conceptual safeguard, PH-LLMs could use partitioned responses for multi-intent prompts that combine permitted and restricted functions. For example, when a user submits recent laboratory results together with a request for lifestyle coaching, the system may provide general lifestyle education while declining to interpret the laboratory findings or tailor clinical recommendations based on them, redirecting interpretation to a qualified clinician. However, partitioned responses are technically fragile if implemented only at the level of response wording. Because LLMs may condition on the broader prompt context, restricted contextual information, such as laboratory values, may implicitly influence the permitted portion of a response. Robust guardrail engineering is therefore needed to reduce context bleed and prevent implicit diagnostic integration [18,22]. Such safeguards may include intent classification, restricted-context masking or separation, output-level verification, and escalation rules when permitted coaching cannot be reliably separated from clinician-dependent interpretation. Safeguard effectiveness should be evaluated through postdeployment monitoring of both safety outcomes, such as appropriate escalation or referral, and engagement outcomes, such as abandonment following boundary prompts [3]. Illustrative scope boundaries are provided in Multimedia Appendix 6.

### Postdeployment Oversight and Version Governance

Predeployment evaluation is necessary but insufficient for PH-LLMs because system behavior, safety performance, and deployment context may change over time. Governance should therefore include structured postdeployment monitoring, low-friction adverse-event reporting, transparency mechanisms, and version-aware change control [94,100,108,134]. The system provides users, caregivers, and clinicians with standardized reporting pathways for clinically relevant events, including dangerous hallucinations, crisis mismanagement, and privacy breaches, adapted to AI-specific harms [21,89,134,135].

As a conceptual response framework, critical adverse events trigger graduated action according to scope and severity, ranging from session-level termination of an affected thread to feature-level disabling of implicated functionality or, when risk is systemic, global suspension of the application. User lockout without alternative crisis resources must be avoided. Developers should publish periodic transparency reports covering adverse events by severity, crisis-protocol activation and outcomes, bias-audit findings and remediation, and model updates with associated safety evaluations [93,102].

For higher-reach deployments, these mechanisms may be supplemented by independent safety oversight funded through structures designed to preserve independence from reviewed entities. As a normative governance proposal, potential models include pooled industry levies administered by neutral standards bodies, regulatory fees, public funding with statutory protections, or coalition-based subscription models with disclosed funding sources and recusal rules [74,81,82,95]. These oversight mechanisms differ in target audience and implementation pathway. Developers and deployers can directly implement technical guardrails, reporting channels, transparency reports, and internal safety audits. By contrast, pooled industry levies, regulatory fees, and publicly funded oversight structures are directed primarily to policymakers, regulators, and standards-setting bodies because they generally require statutory or regulatory authorization. Voluntary coalition-based models may be more immediately feasible, but still require conflict-of-interest safeguards, transparent funding disclosure, and recusal rules to preserve independence.

Postdeployment governance should also be version-aware. Minor releases undergo documented internal validation, whereas major changes, including model updates, prompt-template revisions, plugin integration, interface redesign, or new data-linking functions, may warrant independent reassessment and, where appropriate, recertification [94,100,108,134]. Each release generates auditable change logs and predefined monitoring indicators to support rollback, incident investigation, and detection of behavioral drift after updates [106,108,134]. Illustrative adverse-event severity categories and response pathways are provided in Multimedia Appendix 7.

### Scope and Feasibility

This framework is grounded primarily in text-based, platform-mediated, direct-to-consumer PH-LLM deployment. Multimodal systems introduce additional governance challenges, including biometric-data protection, modality-specific failure modes, and cross-modal inference of sensitive attributes, and therefore require modality-specific validation and corresponding adjustments to consent, accountability, and monitoring structures [12,136]. Feasibility also varies across institutions, jurisdictions, and developer types because certification, audits, postdeployment surveillance, and incident reporting depend on unevenly distributed financial, technical, regulatory, and workforce capacity [100,101].

As the YouthPH-LLM case illustrates, youth-facing PH-LLMs expose an additional limitation of current digital privacy-law frameworks when applied to adolescent mental health support. Age-of-consent and parental authorization requirements, such as those under the General Data Protection Regulation [87], may protect minors’ privacy and parental rights, but strict implementation can conflict with the ethical goal of providing confidential, low-friction access to adolescent mental health support. Routine parental disclosure or burdensome consent workflows may deter help-seeking, particularly among adolescents experiencing psychological distress, stigma, family conflict, or safety concerns [137]. Future governance should therefore distinguish ordinary data-processing consent from immediate safety-oriented support, preserving rapid access to crisis resources while developing jurisdiction-specific mechanisms for age-appropriate privacy, parental involvement, and safeguarding obligations.

The framework should balance underregulation, which may allow safety-critical failures to propagate at scale and shift risk onto vulnerable users, against overregulation, which may consolidate markets around large incumbents, reduce innovation for underrepresented groups, and divert resources from substantive safety work to compliance documentation. Because validated PH-LLM audit and certification cost data are not yet available, cost implications are described as qualitative categories: lower-cost safeguards include identity disclosure, scope labeling, basic adverse-event reporting, and crisis-resource signposting, whereas independent audits, red-team testing, certification preparation, continuous monitoring, and independent safety oversight are likely to impose moderate to high recurring costs. Large technology platforms may absorb these recurring costs, whereas startups, academic deployers, hospitals, and resource-constrained settings may face substantial barriers without proportionate pathways such as regulatory sandboxes, shared evaluation infrastructure, audit reciprocity, market-access incentives, and tiered certification with reduced fixed costs for smaller deployers [138,139].

We distinguish near-term feasible actions, such as identity disclosure and scope labeling, Tier 3 crisis exclusion from training data, and standardized adverse-event reporting, from longer-term aspirational elements, such as harmonized certification reciprocity, sustainably funded independent safety advisory boards, and population-level longitudinal outcomes monitoring. Qualitative cost categories, institutional capacity profiles, and implementation trade-offs are detailed in Multimedia Appendix 8.

## Current Evidence Base and Limitations

The evidence base for ethical and governance concerns in PH-LLMs remains heterogeneous. Established empirical findings include hallucinations and clinically significant omissions in health-related LLM tasks, persistent demographic bias, privacy and confidentiality concerns in direct-to-consumer digital health settings, and failures of some conversational systems to manage crisis-related interactions safely [21,28,38]. Automation bias in PH-LLM contexts is supported mainly by adjacent-domain evidence from clinical decision-support systems, with limited direct PH-LLM evidence [32]. Emerging signals include increasing user reliance on PH-LLMs for health-related guidance, emotionally dependent use patterns, early indicators of therapeutic displacement, and the influence of interface design and deployment context on safety, trust, and care-seeking behavior [53,56-59]. Forward-looking risks include population-level safety effects, large-scale longitudinal profiling, governance challenges in multimodal and open-source deployments, and health-related use of general-purpose LLMs, where evidence remains limited and many safeguards have not yet been prospectively validated. This framework should therefore be understood as evidence-informed but partly anticipatory, identifying plausible governance needs where empirical certainty remains uneven.

## Conclusion

PH-LLMs are rapidly emerging as widely used interfaces for health sensemaking and self-management. Their direct-to-consumer deployment introduces a distinct ethical risk profile spanning 6 domains: privacy, accuracy, equity, transparency, human-AI interaction, and regulatory governance. Because failures across these domains may propagate harm at scale, governance for PH-LLMs should be not only principle-based but also operational and life cycle-oriented. We therefore propose a principlism-grounded framework that translates beneficence, nonmaleficence, autonomy, and justice into deployable safeguards across developers, deployers, health care institutions, regulators, and users. This framework is intended as an evidence-informed but partly anticipatory governance approach rather than an empirically validated solution set. Ongoing oversight will likely require continuous monitoring, adverse-event reporting, and, where appropriate, independent safety evaluation. Future work should prospectively examine the feasibility, effects, and implementation trade-offs of these mechanisms, including whether privacy-preserving forms of health-context activation could support limited adaptation of selected framework components to general-purpose LLMs used for health-related queries.

## Data Availability

Data sharing is not applicable to this article as no new data were created or analyzed in this study.

## Appendix

Multimedia Appendix 1 Protected-attribute handling for fairness monitoring.

Multimedia Appendix 2 Developer lifecycle responsibilities and adverse-event reporting.

Multimedia Appendix 3 Standards and institutional anchors for risk-tiered certification.

Multimedia Appendix 4 YouthPH-LLM case: full stakeholder specification.

Multimedia Appendix 5 Illustrative retention windows for tiered consent architecture.

Multimedia Appendix 6 Illustrative scope boundaries: permitted versus clinician-dependent functions.

Multimedia Appendix 7 Adverse-event severity categories and response pathways.

Multimedia Appendix 8 Feasibility, cost categories, and implementation trade-offs.

## Abbreviations

AI: artificial intelligence
LLM: large language model
PH-LLM: personal health large language model
