# Supplementary material for: Ethical Considerations in Personal Health Large Language Models
Source: J Med Internet Res. 2026 Jun 17;28:e92240. doi: 10.2196/92240 (PMC13324317; doi:10.2196/92240)
Supplement: Multimedia Appendix 1 [file jmir_v28i1e92240_app1.docx]

**Multimedia Appendices**

**Multimedia Appendix 1.**

**Protected-Attribute Handling for Fairness Monitoring**

This appendix specifies how protected attributes should be handled during fairness monitoring of personal health large language models (PH-LLMs), and how disparity findings should translate into audit and remediation actions.

**Attributes monitored**

Fairness audits should examine group-stratified outcomes across demographic and clinically relevant subgroup dimensions, including age, sex assigned at birth, gender identity, race, ethnicity, primary language, disability status, and socioeconomic indicators, where legally permissible and ethically justified. Sexual orientation and immigration status may warrant inclusion only under enhanced privacy and safety safeguards, including encrypted disaggregation, strict role-based access, access logs reviewable by an external ethics body, and prohibition of any operational use beyond aggregate disparity estimation. Decisions to monitor these more sensitive attributes should be made in consultation with affected community representatives. Intersectional combinations, such as language by age or race by gender, should be examined whenever subgroup sample sizes permit reliable estimation.

**Data sources for stratification**

Three data sources are appropriate, in descending order of preference: (1) attributes voluntarily disclosed by users with explicit fairness-audit consent and stored separately from interaction content under access control; (2) synthetic counterfactual variation in matched clinical vignettes constructed for predeployment fairness evaluation; and (3) inferred attributes derived from interaction features, used only for aggregate population-level disparity estimation and never linked to individual user profiles. Inferred attributes should not be used to personalize content, restrict service, determine access, or trigger individual-level interventions. Because protected-class status is often unobserved in operational datasets, fairness assessment based on proxy or inferred attributes is subject to measurement error, partial identification, and group-varying uncertainty [1,2]. Disparity estimates derived from inference should therefore be reported with confidence intervals or uncertainty ranges reflecting both sampling and inference uncertainty, and should not be used as the sole basis for remediation decisions.

**Storage and access controls**

Protected-attribute data should be siloed from operational PH-LLM systems, accessible only to designated fairness audit personnel under documented role-based access, and subject to retention limits proportional to audit cycles. Re-identification risk assessments should accompany any release of stratified audit data outside the audit team. When feasible, privacy-preserving aggregation, minimum cell-size rules, and external audit review should be used to reduce re-identification risks, especially for small or intersectional subgroups.

**Disparity triggers**

The trigger framework is informed by health AI transparency, evaluation, LLM-specific reporting, and lifecycle governance guidance, including the STANdards for data Diversity, INclusivity and Generalisability (STANDING Together) consensus recommendations for dataset transparency [3], the DECIDE-AI reporting guideline for early-stage evaluation of AI-driven decision support systems [4], the TRIPOD-LLM reporting guideline for studies using LLMs [5], and the FUTURE-AI international consensus guideline for trustworthy and deployable AI in healthcare [6]. It also uses the US Equal Employment Opportunity Commission (EEOC) four-fifths rule [7] only as an analogy for screening-level relative disparity detection, rather than as a validated health-AI threshold. Predefined thresholds should function as screening triggers for further audit, contextual review, and confirmatory evaluation rather than as definitive evidence of discrimination or model bias. Illustrative triggers include: (1) group-stratified rates of unsafe guidance differing by more than a prespecified margin, such as a two-percentage-point absolute difference or a relative risk above 1.25, relative to a prespecified reference group or population baseline and among groups with sufficient sample size; (2) group-stratified crisis-escalation success rates falling below the population baseline by a prespecified margin; (3) directional bias detected in counterfactual vignette evaluation, such as systematically lower rates of urgent-care escalation guidance or systematically more frequent redirection to clinicians for routine information when otherwise identical vignettes vary only in race or ethnicity; and (4) accessibility failures concentrated in a specific language group or assistive-technology user cohort.

**Remediation pathway**

Following trigger activation and contextual review, a remediation pathway may include root-cause analysis of training data composition, retrieval-source coverage, prompt-handling logic, model-update history, and interface design; targeted mitigation, such as retrieval-source rebalancing, prompt or guardrail revision, model or policy adjustment, or interface modification; revalidation against the original disparity metric and adjacent fairness measures to identify possible compensatory shifts; and disclosure of the audit finding, mitigation action, and revalidation outcome in the next periodic transparency report. Persistent disparities that remain after reasonable mitigation efforts may warrant escalation to the relevant oversight body and, where the affected functionality poses material safety, access, or equity risks, temporary feature restriction or suspension pending further review. Because different fairness criteria, such as demographic parity, error-rate balance, and calibration, cannot generally be optimized simultaneously [8], audit protocols should pre-specify the fairness criterion most relevant to the deployment context, document the rationale, and revisit this choice when the population, use case, or deployment setting changes.

**References**

1. Elliott MN, Morrison PA, Fremont A, McCaffrey DF, Pantoja P, Lurie N. Using the Census Bureau's surname list to improve estimates of race/ethnicity and associated disparities. Health Serv Outcomes Res Methodol. 2009;9(2):69-83. doi:10.1007/s10742-009-0047-1
2. Kallus N, Mao X, Zhou A. Assessing algorithmic fairness with unobserved protected class using data combination. Manage Sci. 2022;68(3):1959-1981. doi:10.1287/mnsc.2020.3850
3. Alderman JE, Palmer J, Laws E, McCradden MD, Ordish J, Ghassemi M, et al. Tackling algorithmic bias and promoting transparency in health datasets: the STANDING Together consensus recommendations. Lancet Digit Health. 2025;7(1):e64-e88. doi:10.1016/S2589-7500(24)00224-3
4. Vasey B, Nagendran M, Campbell B, Clifton DA, Collins GS, Denaxas S, et al. Reporting guideline for the early-stage clinical evaluation of decision support systems driven by artificial intelligence: DECIDE-AI. Nat Med. 2022;28(5):924-933. doi:10.1038/s41591-022-01772-9
5. Gallifant J, Afshar M, Ameen S, Aphinyanaphongs Y, Chen S, Cacciamani G, et al. The TRIPOD-LLM reporting guideline for studies using large language models. Nat Med. 2025;31(1):60-69. doi:10.1038/s41591-024-03425-5
6. Lekadir K, Frangi AF, Porras AR, Glocker B, Cintas C, Langlotz CP, et al. FUTURE-AI: international consensus guideline for trustworthy and deployable artificial intelligence in healthcare. BMJ. 2025;388:e081554. doi:10.1136/bmj-2024-081554
7. US Equal Employment Opportunity Commission. Uniform Guidelines on Employee Selection Procedures. 29 CFR §1607.4(D). 1978. https://www.ecfr.gov/current/title-29/subtitle-B/chapter-XIV/part-1607. [accessed 2026-04-10]
8. Chouldechova A. Fair prediction with disparate impact: a study of bias in recidivism prediction instruments. Big Data. 2017;5(2):153-163. doi:10.1089/big.2016.0047
