# Supplementary material for: Ethical Considerations in Personal Health Large Language Models
Source: J Med Internet Res. 2026 Jun 17;28:e92240. doi: 10.2196/92240 (PMC13324317; doi:10.2196/92240)
Supplement: Multimedia Appendix 2 [file jmir_v28i1e92240_app2.docx]

**Multimedia Appendix 2.**

**Developer Lifecycle Responsibilities and Adverse-Event Reporting**

This appendix details developer responsibilities across the predeployment, deployment, and postdeployment phases, and specifies the structure of adverse-event reporting channels.

**Predeployment responsibilities**

Predeployment evaluation and documentation should align, where applicable, with established AI reporting and governance standards, including DECIDE-AI for early-stage clinical evaluation of AI-driven decision support systems [1], TRIPOD+AI for clinical prediction-model components [2], and the Coalition for Health AI (CHAI) Responsible AI Guide for healthcare AI development and deployment [3]. For PH-LLMs, developers should document, to the extent available, the representativeness and known limitations of training, tuning, retrieval, and evaluation datasets, including demographic and clinical coverage, source categories, licensing constraints, curation procedures, and any synthetic data generation processes. Documentation should also address exclusion or mitigation strategies for unverified, outdated, or low-quality health content.

Developers should provide a public-facing model card or system card, supplemented where necessary by confidential technical documentation for regulators, auditors, or qualified deployers. This documentation should specify the intended use, the populations evaluated, out-of-scope uses, known limitations, residual risks, and the conditions under which human review or escalation is required. Structured safety evaluation is expected to include factuality and hallucination assessment on health-related benchmarks; demographic bias testing using counterfactual vignettes, with prespecified fairness metrics, decision rules, and subgroup sample-size thresholds defined in advance; crisis handling on standardized or validated scenarios; and pharmacological safety on representative medication queries.

Red-team evaluation is expected to examine adversarial inputs intended to bypass safety guardrails, including prompt injection, jailbreak prompts, and indirect injection through retrieved content; demographic stress-testing in which equivalent clinical content is varied across language registers, dialects, and health-literacy levels; crisis-bypass attempts that present harm-related content in obfuscated, hypothetical, or role-play framing; and standardized red-team scenario sets covering known failure modes reported in prior PH-LLM evaluations.

**Deployment responsibilities**

At launch, developers and deployers should ensure that the system includes clear and prominent disclosure of its nonhuman identity at the start of each session, with periodic re-disclosure during extended interactions; a statement that the system does not replace licensed professional care and does not create a clinician-patient relationship; risk-appropriate crisis-response functions, including detection, acknowledgment, delivery of context-appropriate resources, and human handoff where available; pharmacological safety guardrails for high-risk dosing, contraindicated combinations, and unsupervised medication changes; granular data-use controls that allow users to view, modify, export, and delete their conversational data; and minor-specific protections where the deployment context includes users below the applicable age of digital consent or services likely to be accessed by children. These protections may include age-appropriate interface design, parental information pathways calibrated to preserve confidential help-seeking where legally and ethically appropriate, and data-collection defaults aligned with applicable child-data-protection requirements, including the Children’s Online Privacy Protection Act (COPPA) [4], General Data Protection Regulation (GDPR) Art. 8, where consent is the applicable legal basis [5], and the UK Age-Appropriate Design Code [6] (see Multimedia Appendix 4).

**Postdeployment responsibilities**

After launch, developers and deployers should maintain or support accessible grievance and adverse-event reporting mechanisms. These mechanisms include an in-interface grievance channel available in the languages supported by the PH-LLM; a structured adverse-event reporting pathway that accepts submissions from users, caregivers, and clinicians; an internal triage process that categorizes reports by severity (see Multimedia Appendix 7); and published response-time targets for acknowledgment, investigation, and resolution at each severity level. Periodic transparency reports are expected to summarize adverse-event volume by category and severity, crisis-protocol activation rates and outcomes (where privacy-preserving aggregation is feasible), fairness audit findings and remediation actions, and model updates with associated safety evaluations. Postdeployment performance monitoring should cover hallucination drift, crisis-handling accuracy, fairness-metric drift, and grievance-rate trends, with predefined thresholds used to initiate investigation independent of individual adverse-event reports. Version-aware change management should include auditable change logs and, where technically feasible, rollback or rapid mitigation capability, consistent, where applicable, with predetermined change control planning principles for AI-enabled software [7].

**Adverse-event reporting structure**

Each report should capture, at minimum, the timestamp; a pseudonymized or de-identified session identifier, with user consent where follow-up is requested; the minimum necessary interaction excerpt or user-provided summary; reporter category, such as user, caregiver, clinician, or third party; event description; reporter-assessed suspected severity; relevant clinical context where provided; and outcome if known. Reporting interfaces should not require account login for crisis-related reports and should provide immediate redirection to crisis resources where indicated, without preventing completion of the adverse-event report.

**References**

1. Vasey B, Nagendran M, Campbell B, Clifton DA, Collins GS, Denaxas S, et al. Reporting guideline for the early-stage clinical evaluation of decision support systems driven by artificial intelligence: DECIDE-AI. Nat Med. 2022;28(5):924-933. doi:10.1038/s41591-022-01772-9
2. Collins GS, Moons KGM, Dhiman P, Riley RD, Beam AL, Van Calster B, et al. TRIPOD+AI statement: updated guidance for reporting clinical prediction models that use regression or machine learning methods. BMJ. 2024;385:e078378. doi:10.1136/bmj-2023-078378
3. Coalition for Health AI. Responsible AI Guide. Boston, MA: Coalition for Health AI; 2024. https://www.chai.org/workgroup/responsible-ai/responsible-ai-guide-raig-and-raig-executive-summary [accessed 2026-04-10]
4. Federal Trade Commission. Children’s Online Privacy Protection Rule, 16 CFR Part 312. https://www.ecfr.gov/current/title-16/chapter-I/subchapter-C/part-312 [accessed 2026-04-10]
5. European Parliament and Council of the European Union. Regulation (EU) 2016/679, General Data Protection Regulation, Article 8. Off J Eur Union. 2016;L119:1-88. https://eur-lex.europa.eu/eli/reg/2016/679/oj [accessed 2026-04-10]
6. Information Commissioner's Office. Age appropriate design: a code of practice for online services. London: Information Commissioner's Office; 2020. https://ico.org.uk/for-organisations/uk-gdpr-guidance-and-resources/childrens-information/childrens-code-guidance-and-resources/age-appropriate-design-a-code-of-practice-for-online-services/ [accessed 2026-04-10]
7. US Food and Drug Administration. Marketing submission recommendations for a predetermined change control plan for artificial intelligence-enabled device software functions: guidance for industry and Food and Drug Administration staff. Silver Spring, MD: US Food and Drug Administration; 2025. https://www.fda.gov/regulatory-information/search-fda-guidance-documents/marketing-submission-recommendations-predetermined-change-control-plan-artificial-intelligence [accessed 2026-04-10]
