# Supplementary material for: Ethical Considerations in Personal Health Large Language Models
Source: J Med Internet Res. 2026 Jun 17;28:e92240. doi: 10.2196/92240 (PMC13324317; doi:10.2196/92240)
Supplement: Multimedia Appendix 3 [file jmir_v28i1e92240_app3.docx]

**Multimedia Appendix 3.**

**Standards and Institutional Anchors for Risk-Tiered Certification**

This appendix lists the principal international and national instruments that should serve as reference standards for PH-LLM certification, organized by domain.

**General AI governance**

Organisation for Economic Co-operation and Development (OECD) Recommendation of the Council on Artificial Intelligence (2019, amended 2024) [1]; National Institute of Standards and Technology (NIST) Artificial Intelligence Risk Management Framework 1.0 (2023) [2]; European Union Regulation (EU) 2024/1689, Artificial Intelligence Act (AI Act) [3]; and national AI governance instruments such as the Republic of Korea Framework Act on the Development of Artificial Intelligence and the Creation of a Foundation for Trust (promulgated 2025; effective 2026) [4], the People’s Republic of China Interim Measures for the Management of Generative AI Services (2023) [5], Japan AI Guidelines for Business, Version 1.1 (2025) [6], and Singapore Model AI Governance Framework for Generative AI (2024) [7].

**Health-specific AI governance**

WHO Ethics and Governance of AI for Health: Guidance on Large Multi-Modal Models (2025) [8]; WHO Ethics and Governance of AI for Health (2021) [9]; WHO Regulatory Considerations on AI for Health (2023) [10]; FDA, Health Canada, and MHRA Good Machine Learning Practice Guiding Principles (2021) [11]; FDA guidance on Predetermined Change Control Plans for AI-enabled device software functions (2025) [12]; FDA Clinical Decision Support Software guidance (2022; updated 2026) [13]; Coalition for Health AI Responsible AI Guide (RAIG) (2024) [14].

**Privacy and data protection**

EU General Data Protection Regulation [15]; UK GDPR special category data guidance issued by the Information Commissioner's Office [16]; US HIPAA framework [17], including HHS guidance on online tracking technologies [18] and on the individual access right for health apps and APIs [19]; jurisdiction-specific consumer health data laws; and minor-specific protection frameworks including the US Children’s Online Privacy Protection Act [20], GDPR Article 8 digital consent provisions [15], and the UK Age-Appropriate Design Code [21] where the deployment serves users below the age of digital consent.

**Bias, fairness, and transparency**

STANDING Together consensus recommendations on dataset transparency [22]; DECIDE-AI reporting guideline for early-stage clinical evaluation of AI decision support [23]; TRIPOD+AI statement for clinical prediction model reporting [24]; CONSORT-AI [25] and SPIRIT-AI [26] for reporting and protocols of clinical trials evaluating AI interventions; Web Content Accessibility Guidelines 2.2 [27]; and the US Centers for Disease Control and Prevention health literacy guidance [28].

**Safety and pharmacovigilance**

WHO pharmacovigilance principles [29]; FDA Adverse Event Reporting System (FAERS) framework [30]; CIOMS Working Group XIV report on Artificial Intelligence in Pharmacovigilance (2025) [31]; SAMHSA National Guidelines for a Behavioral Health Coordinated System of Crisis Care [32].

**Application by certification tier**

Baseline tier deployments should demonstrate alignment with WHO guidance on ethics and governance of AI for health and applicable jurisdictional privacy and consumer-protection law. Mid-tier deployments should additionally demonstrate alignment with NIST AI RMF, relevant clinical evaluation reporting standards (DECIDE-AI, TRIPOD+AI, CONSORT-AI as applicable), and dataset transparency standards (STANDING Together). High-tier deployments handling crisis-prone populations, minors, or medication guidance should additionally demonstrate alignment with applicable medical device frameworks (EU AI Act high-risk obligations, FDA software-as-a-medical-device guidance), pharmacovigilance principles (WHO, CIOMS Working Group XIV), minor-protection frameworks where applicable, and crisis-care standards (SAMHSA).

**References**

1. Organisation for Economic Co-operation and Development. Recommendation of the Council on Artificial Intelligence. OECD/LEGAL/0449. Paris: OECD; 2019 (amended 2024). https://legalinstruments.oecd.org/en/instruments/oecd-legal-0449 [accessed 2026-04-10]
2. National Institute of Standards and Technology. Artificial Intelligence Risk Management Framework (AI RMF 1.0). Gaithersburg, MD: US Department of Commerce; 2023. doi:10.6028/NIST.AI.100-1
3. Regulation (EU) 2024/1689 of the European Parliament and of the Council of 13 June 2024 laying down harmonised rules on artificial intelligence (Artificial Intelligence Act). Off J Eur Union. 2024;L 2024/1689. https://eur-lex.europa.eu/eli/reg/2024/1689/oj [accessed 2026-04-10]
4. Republic of Korea. Framework Act on the Development of Artificial Intelligence and Establishment of Trust Foundation. Law No. 20676, enacted January 21, 2025; effective January 22, 2026. https://www.law.go.kr [accessed 2026-04-10]
5. Cyberspace Administration of China. Interim Measures for the Management of Generative Artificial Intelligence Services. Beijing: Cyberspace Administration of China; 2023. http://www.cac.gov.cn/2023-07/13/c_1690898327029107.htm [accessed 2026-04-10]
6. Ministry of Economy, Trade and Industry; Ministry of Internal Affairs and Communications. AI Guidelines for Business Version 1.0. Tokyo: Government of Japan; 2024. https://www.meti.go.jp/english/press/2024/0419_002.html [accessed 2026-04-10]
7. Infocomm Media Development Authority; Personal Data Protection Commission. Model AI Governance Framework for Generative AI. Singapore: IMDA/PDPC; 2024. https://aiverifyfoundation.sg/resources/model-ai-governance-framework-genai/ [accessed 2026-04-10]
8. World Health Organization. Ethics and governance of artificial intelligence for health: Guidance on large multi-modal models. Geneva: WHO; 2025. https://www.who.int/publications/i/item/9789240084759 [accessed 2026-04-10]
9. World Health Organization. Ethics and governance of artificial intelligence for health: WHO guidance. Geneva: WHO; 2021. https://www.who.int/publications/i/item/9789240029200 [accessed 2026-04-10]
10. World Health Organization. Regulatory considerations on artificial intelligence for health. Geneva: WHO; 2023. https://www.who.int/publications/i/item/9789240078871 [accessed 2026-04-10]
11. US Food and Drug Administration; Health Canada; UK Medicines and Healthcare products Regulatory Agency. Good Machine Learning Practice for Medical Device Development: Guiding Principles. 2021. https://www.fda.gov/medical-devices/software-medical-device-samd/good-machine-learning-practice-medical-device-development-guiding-principles [accessed 2026-04-10]
12. US Food and Drug Administration. Marketing submission recommendations for a predetermined change control plan for artificial intelligence-enabled device software functions: guidance for industry and Food and Drug Administration staff. Silver Spring, MD: US Food and Drug Administration; 2025. https://www.fda.gov/regulatory-information/search-fda-guidance-documents/marketing-submission-recommendations-predetermined-change-control-plan-artificial-intelligence [accessed 2026-04-10]
13. US Food and Drug Administration. Clinical Decision Support Software: guidance for industry and Food and Drug Administration staff. Silver Spring, MD: US Food and Drug Administration; 2022 (updated 2026). https://www.fda.gov/regulatory-information/search-fda-guidance-documents/clinical-decision-support-software [accessed 2026-04-10]
14. Coalition for Health AI. Responsible AI Guide (RAIG). Boston, MA: Coalition for Health AI; 2024. https://www.chai.org/workgroup/responsible-ai/responsible-ai-guide-raig-and-raig-executive-summary [accessed 2026-04-10]
15. Regulation (EU) 2016/679 of the European Parliament and of the Council of 27 April 2016 on the protection of natural persons with regard to the processing of personal data and on the free movement of such data (General Data Protection Regulation). Off J Eur Union. 2016;L119:1-88. https://eur-lex.europa.eu/eli/reg/2016/679/oj [accessed 2026-04-10]
16. Information Commissioner's Office. Special category data: guidance on the UK GDPR. Wilmslow, UK: Information Commissioner's Office. https://ico.org.uk/for-organisations/uk-gdpr-guidance-and-resources/lawful-basis/special-category-data/ [accessed 2026-04-10]
17. US Department of Health and Human Services. Health Insurance Portability and Accountability Act of 1996, Public Law 104-191. Privacy and Security Rules codified at 45 CFR Parts 160, 162, and 164. https://www.hhs.gov/hipaa/index.html [accessed 2026-04-10]
18. US Department of Health and Human Services, Office for Civil Rights. Use of online tracking technologies by HIPAA covered entities and business associates. Washington, DC: HHS; 2024. https://www.hhs.gov/hipaa/for-professionals/privacy/guidance/hipaa-online-tracking/index.html [accessed 2026-04-10]
19. US Department of Health and Human Services, Office for Civil Rights. Individuals' right under HIPAA to access their health information: guidance on access for third-party health applications and APIs. Washington, DC: HHS. https://www.hhs.gov/hipaa/for-professionals/privacy/guidance/access/index.html [accessed 2026-04-10]
20. Children's Online Privacy Protection Act of 1998, 15 USC §6501-6506. Implementing rule at 16 CFR Part 312. https://www.ecfr.gov/current/title-16/chapter-I/subchapter-C/part-312 [accessed 2026-04-10]
21. Information Commissioner's Office. Age appropriate design: a code of practice for online services. London: Information Commissioner's Office; 2020. https://ico.org.uk/for-organisations/uk-gdpr-guidance-and-resources/childrens-information/childrens-code-guidance-and-resources/age-appropriate-design-a-code-of-practice-for-online-services/ [accessed 2026-04-10]
22. Alderman JE, Palmer J, Laws E, McCradden MD, Ordish J, Ghassemi M, et al. Tackling algorithmic bias and promoting transparency in health datasets: the STANDING Together consensus recommendations. Lancet Digit Health. 2025;7(1):e64-e88. doi:10.1016/S2589-7500(24)00224-3
23. Vasey B, Nagendran M, Campbell B, Clifton DA, Collins GS, Denaxas S, et al. Reporting guideline for the early-stage clinical evaluation of decision support systems driven by artificial intelligence: DECIDE-AI. Nat Med. 2022;28(5):924-933. doi:10.1038/s41591-022-01772-9
24. Collins GS, Moons KGM, Dhiman P, Riley RD, Beam AL, Van Calster B, et al. TRIPOD+AI statement: updated guidance for reporting clinical prediction models that use regression or machine learning methods. BMJ. 2024;385:e078378. doi:10.1136/bmj-2023-078378
25. Liu X, Cruz Rivera S, Moher D, Calvert MJ, Denniston AK; SPIRIT-AI and CONSORT-AI Working Group. Reporting guidelines for clinical trial reports for interventions involving artificial intelligence: the CONSORT-AI extension. Nat Med. 2020;26(9):1364-1374. doi:10.1038/s41591-020-1034-x
26. Cruz Rivera S, Liu X, Chan AW, Denniston AK, Calvert MJ; SPIRIT-AI and CONSORT-AI Working Group. Guidelines for clinical trial protocols for interventions involving artificial intelligence: the SPIRIT-AI extension. Nat Med. 2020;26(9):1351-1363. doi:10.1038/s41591-020-1037-7
27. World Wide Web Consortium. Web Content Accessibility Guidelines (WCAG) 2.2. W3C Recommendation. 2023. https://www.w3.org/TR/WCAG22/ [accessed 2026-04-10]
28. Centers for Disease Control and Prevention. Health Literacy. Atlanta, GA: US Department of Health and Human Services. https://www.cdc.gov/health-literacy/ [accessed 2026-04-10]
29. World Health Organization. The importance of pharmacovigilance: safety monitoring of medicinal products. Geneva: WHO; 2002. https://iris.who.int/handle/10665/42493 [accessed 2026-04-10]
30. US Food and Drug Administration. FDA Adverse Event Reporting System (FAERS). Silver Spring, MD: US Food and Drug Administration. https://www.fda.gov/drugs/questions-and-answers-fdas-adverse-event-reporting-system-faers/fda-adverse-event-reporting-system-faers-public-dashboard [accessed 2026-04-10]
31. Council for International Organizations of Medical Sciences. Artificial Intelligence in Pharmacovigilance: Report of CIOMS Working Group XIV. Geneva: CIOMS; 2025. https://cioms.ch/working_groups/working-group-xiv-artificial-intelligence-in-pharmacovigilance/ [accessed 2026-04-10]
32. Substance Abuse and Mental Health Services Administration. National Guidelines for a Behavioral Health Coordinated System of Crisis Care. Rockville, MD: SAMHSA; 2025. https://www.samhsa.gov/find-help/988/national-guidelines-best-practices [accessed 2026-04-10]
