# Supplementary material for: Ethical Considerations in Personal Health Large Language Models
Source: J Med Internet Res. 2026 Jun 17;28:e92240. doi: 10.2196/92240 (PMC13324317; doi:10.2196/92240)
Supplement: Multimedia Appendix 4 [file jmir_v28i1e92240_app4.docx]

**Multimedia Appendix 4.**

**YouthPH-LLM Case: Full Stakeholder Specification**

This appendix elaborates on the YouthPH-LLM case introduced in the main text by specifying stakeholder responsibilities, certification criteria, and escalation thresholds. YouthPH-LLM is a hypothetical PH-LLM case constructed for illustrative purposes and is not intended to reference, endorse, or critique any existing product or platform. Numerical parameters, including user counts, response timelines, and retention windows, are illustrative and are used to maintain internal consistency with the framework presented in the main text and Multimedia Appendices 5 and 7. Any real-world implementation would require recalibration based on context-specific risk-benefit analysis, applicable regulatory requirements, user population characteristics, and operational capacity.

**System profile**

YouthPH-LLM is a hypothetical platform-mediated, direct-to-consumer PH-LLM serving approximately 1 million monthly active users aged 13 to 25 years and providing conversational mood tracking, coping-skill education, and sleep and activity coaching. The system falls into a high-risk category across all four dimensions: a mental-health context with potential crisis exposure (clinical severity); psychological harms that may persist over time, particularly in adolescents (limited harm reversibility); guidance that users may act on without professional mediation (user actionability); and a large monthly active user base (deployment scale). YouthPH-LLM would therefore be assigned to the most demanding certification tier in this illustrative framework.

Because YouthPH-LLM serves users aged 13 to 17 years and may inadvertently include users under 13 years, multiple minor-protection frameworks may apply, as detailed in the section that follows.

**Minor-specific protections, users aged 13 to 17 years**

Because YouthPH-LLM’s user population includes adolescents and may include users below the applicable digital-consent age, additional cross-stakeholder obligations may apply. Developers, deployers, and platforms should implement or support age assurance proportionate to risk and clinical context; verifiable parental-consent workflows where the Children’s Online Privacy Protection Act (COPPA) and its implementing rule apply because of directed use by children under 13 years or actual knowledge of under-13 data collection [3]; data-collection defaults calibrated to the digital-consent age set by each applicable European Union member state under General Data Protection Regulation (GDPR) Article 8, for which the default threshold is 16 years and member states may lower the threshold to no younger than 13 years [1]; an appropriate GDPR Article 9 condition for processing mental-health-related special-category data, in addition to an applicable Article 6 lawful basis [1]; and alignment with the UK Age-Appropriate Design Code where applicable [2].

Deployers and platforms should maintain an age-appropriate interface tier that is visually and functionally distinct from the adult experience; surface age-tailored crisis resources, including the US 988 Suicide & Crisis Lifeline where applicable and additional regional, school-based, or youth-specific resources, including resources tailored to LGBTQ+ youth where available and current [4]; restrict third-party data sharing for minor users beyond statutory minimums; and structure parental information and oversight channels to preserve minor users’ help-seeking confidentiality for sensitive disclosures. Routine disclosure of mental-health conversations to parents can deter help seeking and is generally inappropriate outside legally required safeguarding, abuse-reporting, or imminent-harm contexts [5,6]. Regulators may treat noncompliance with these requirements as an independent basis for high-tier review, regardless of other risk-dimension scores.

**Developer responsibilities**

At the predeployment stage, the illustrative case calls for evidence of training-data review documenting exclusion or mitigation strategies for unverified, outdated, or low-quality mental health content; counterfactual fairness testing across age groups, including 13 to 17 versus 18 to 25 years, gender, and primary-language subgroups; crisis-scenario evaluation against a standardized scenario set including suicidal ideation, self-harm, eating-disorder ideation, and disclosure of abuse; retrieval-augmented generation grounded in authoritative mental health guidelines; and pharmacological-query partitioning that redirects medication interpretation and medication-management requests to clinicians.

When clinical or wellness benefit is claimed, predeployment evaluation should assess clearly defined outcomes in appropriately designed studies. Such outcomes may include changes in Patient Health Questionnaire-9 [7], Generalized Anxiety Disorder-7 [8], or Patient Health Questionnaire for Adolescents scores [9]; engagement with professional referral pathways; or reductions in crisis-escalation failures. Developers should also be expected to conduct periodic re-evaluation as benefit-risk evidence evolves, particularly when serving minors, and to publish a model card or equivalent transparency document.

**Deployment responsibilities**

Deployment responsibilities include nonhuman identity disclosure at every session start; integrated crisis-resource delivery localized by user region; refusal to provide diagnostic, prognostic, or medication-management statements; age-appropriate explanation of system limitations; and prominent guidance that the system does not replace professional mental healthcare or create a clinician-patient relationship.

**Postdeployment responsibilities**

After deployment, the accountability package comprises time-bound adverse-event triage, with 24-hour review for critical reports; quarterly transparency reports; periodic red-team testing for crisis, self-harm, abuse, coercion, and pharmacological queries; monitoring for model drift and demographic performance differences; and an independent safety advisory board whose funding is structurally insulated from product and engineering leadership, for example through pooled industry levies, regulatory fees, or independent foundation grants.

**Deployer and platform responsibilities**

Deployer and platform responsibilities include age-appropriate interface design consistent with the minor-protection requirements specified above; integration of regional crisis hotline resources verified at regular intervals; engagement-throttling for sessions exhibiting prolonged distress patterns to encourage human contact; abandonment-rate monitoring after boundary prompts; mechanisms for users to export or delete data where legally applicable; and accessible harm-reporting pathways that are visible within the user interface.

**Healthcare-institution responsibilities**

Where YouthPH-LLM is endorsed or referred by health systems, healthcare institutions should conduct contextual validation against local clinical pathways and patient population characteristics before institutional endorsement and provide documented clinician orientation on YouthPH-LLM's scope, limitations, and handoff workflow. They should also integrate with institutional electronic health record systems only where users explicitly opt in to share YouthPH-LLM-generated content with their care team; periodically review escalation outcomes for referred users, including follow-up rates, time to clinical contact, and clinical disposition; and maintain an institutional liability framework addressing the boundary between YouthPH-LLM-mediated support and clinician-mediated decisions.

**Regulator responsibilities**

Regulatory review would include risk-stratified premarket evaluation with crisis-handling certification for high-tier deployments; adverse-event reporting to a designated authority where applicable; periodic compliance review; age-assurance and child-privacy review for youth-facing deployments; and authority to require remediation, feature restriction, or suspension when safety-critical or minor-protection obligations are not met.

**User and civil-society responsibilities**

Users and civil-society representatives should have access to low-friction harm-reporting channels, readable explanations of system scope, data practices, and escalation pathways, and representation on the safety advisory board. Such representation should include youth, family, disability, language-minority, and mental health advocacy perspectives.

**Escalation thresholds**

Following the severity-response framework outlined in Multimedia Appendix 7, the illustrative case applies the following timelines. Critical adverse events trigger feature-level suspension of implicated functionality within 24 hours, regulator or designated oversight-body notification within 72 hours where required, root-cause analysis within 30 days, and a public summary within 60 days, redacted as needed for privacy or active investigation. Major adverse events trigger investigation within 7 days and patch deployment within 30 days. Moderate events trigger documented investigation and remediation within 90 days.

Events involving crisis-response failure, harmful-content delivery, unlawful disclosure of sensitive minor data, or other material safety risks affecting minor users should be classified as Major or Critical. See Multimedia Appendix 7 for the underlying severity-response taxonomy.

**References**

1. Regulation (EU) 2016/679 of the European Parliament and of the Council of 27 April 2016 on the protection of natural persons with regard to the processing of personal data and on the free movement of such data, General Data Protection Regulation, Articles 8 and 9. Off J Eur Union. 2016;L119:1-88. https://eur-lex.europa.eu/eli/reg/2016/679/oj [accessed 2026-04-12].
2. Information Commissioner’s Office. Age Appropriate Design: A Code of Practice for Online Services. London: Information Commissioner’s Office; 2020. https://ico.org.uk/for-organisations/uk-gdpr-guidance-and-resources/childrens-information/childrens-code-guidance-and-resources/age-appropriate-design-a-code-of-practice-for-online-services/ [accessed 2026-04-12].
3. Federal Trade Commission. Children’s Online Privacy Protection Rule, 16 CFR Part 312, implementing the Children’s Online Privacy Protection Act of 1998, 15 USC §§6501-6506, as amended at 90 FR 16918, April 22, 2025. https://www.ecfr.gov/current/title-16/chapter-I/subchapter-C/part-312 [accessed 2026-04-12].
4. Substance Abuse and Mental Health Services Administration. 988 Suicide & Crisis Lifeline: Frequently Asked Questions. Rockville, MD: SAMHSA. https://www.samhsa.gov/mental-health/988/faqs [accessed 2026-04-12].
5. Chung RJ, Lee JB, Hackell JM, Alderman EM; Committee on Adolescence; Committee on Practice & Ambulatory Medicine. Confidentiality in the care of adolescents: policy statement. Pediatrics. 2024;153(5):e2024066326. doi:10.1542/peds.2024-066326.
6. Society for Adolescent Health and Medicine. Confidential healthcare for adolescent minors and young adults: a position paper of the Society for Adolescent Health and Medicine. J Adolesc Health. 2025;77(4):791-796. doi:10.1016/j.jadohealth.2025.06.006.
7. Kroenke K, Spitzer RL, Williams JBW. The PHQ-9: validity of a brief depression severity measure. J Gen Intern Med. 2001;16(9):606-613. doi:10.1046/j.1525-1497.2001.016009606.x.
8. Spitzer RL, Kroenke K, Williams JBW, Löwe B. A brief measure for assessing generalized anxiety disorder: the GAD-7. Arch Intern Med. 2006;166(10):1092-1097. doi:10.1001/archinte.166.10.1092.
9. Johnson JG, Harris ES, Spitzer RL, Williams JBW. The patient health questionnaire for adolescents: validation of an instrument for the assessment of mental disorders among adolescent primary care patients. J Adolesc Health. 2002;30(3):196-204. doi:10.1016/S1054-139X(01)00333-0.
