# Supplementary material for: Ethical Considerations in Personal Health Large Language Models
Source: J Med Internet Res. 2026 Jun 17;28:e92240. doi: 10.2196/92240 (PMC13324317; doi:10.2196/92240)
Supplement: Multimedia Appendix 5 [file jmir_v28i1e92240_app5.docx]

**Multimedia Appendix 5.**

**Illustrative Retention Windows for Tiered Consent Architecture**

This appendix provides illustrative retention parameters for the four-tier consent architecture introduced in Table 3 of the main text. These values are illustrative defaults to support implementation planning. Specific durations should be recalibrated through documented risk-benefit analysis accounting for clinical utility, re-identification risk, user-preference evidence, technical necessity, and applicable jurisdictional law. Relevant legal and governance considerations include data minimization principles, lawful-basis requirements, and rights of access and erasure under the GDPR Articles 5, 6, 15, and 17 [1], HIPAA access rights to designated record sets and Privacy Rule documentation-retention requirements where applicable [2], and privacy risk-management principles such as the National Institute of Standards and Technology (NIST) Privacy Framework [3].

Table 5-1. Illustrative retention parameters by content tier

| Tier | Content category | Default retention | Re-consent cadence | Third-party sharing default | User-requested deletion fulfillment timeline |
| --- | --- | --- | --- | --- | --- |
| Tier 1 | General health queries (symptom information, wellness questions, lifestyle education) | 90 days default, user-adjustable from immediate deletion to 365 days, calibrated to data minimization principles under applicable privacy law [1] | Annual prompt for preference review; explicit re-consent triggered by material policy changes | No third-party sharing by default; aggregated or de-identified research use only under valid legal basis [1,3] | Within 30 days, unless applicable law requires earlier action |
| Tier 2 | Mental health disclosures (mood, stress, relationship distress, non-crisis emotional content) | 30 days by default; immediate deletion selectable. Aggregated de-identified audit features may be retained separately for the audit cycle. | Re-consent at each new session disclosing Tier 2 content | Prohibited for advertising, product personalization, or unrelated operational use; safety audits limited to privacy-preserving analytics | Within 7 days, unless legal, safety, or audit obligations require a limited deferral |
| Tier 3 | Crisis-related content (self-harm, suicidality, interpersonal violence, acute psychiatric emergencies) | Purged within 7 days following completion of any required safety audit; otherwise not retained beyond the immediate safety response | Not applicable; consent prompts replaced by immediate safety response | Limited to legally mandated emergency intervention in imminent-harm contexts | Immediate acknowledgment; deletion as soon as legally permissible, with deferral only for required safety, audit, investigation, or safeguarding retention |
| Tier 4 | Longitudinal narratives (multi-session aggregated profiles, derived behavioral patterns) | 6 months from last interaction; user-controlled deletion at any time | Re-consent every 6 months with summary of derived insights | Default opt-out; explicit per-purpose authorization required for any sharing | Within 7 days, including derived features or aggregates where technically feasible and legally required |

**Tier 4 onset**

Tier 4 classification should activate when either of the following conditions is met: (a) interactions span at least 3 sessions over more than 14 days, or (b) the system computes any derived behavioral feature from cross-session aggregation, whichever occurs first. Users should receive notice at Tier 4 activation and be asked for explicit re-consent before longitudinal profiling or derived behavioral features are retained beyond the default period.

**Multi-tier interactions**

Where a single interaction contains content spanning multiple tiers, tier classification is performed at the content level rather than the session level. This approach prevents non-crisis content within a Tier 3-flagged session from being automatically subjected to Tier 3 immediate-purge rules unless that content is safety-relevant. The highest applicable tier governs retention treatment for each content unit, while non-Tier 3 content within a mixed-tier session retains its native-tier treatment.

**Tier 3 audit-period handling**

Crisis-related content subject to mandatory safety, audit, investigation, or safeguarding retention should be encrypted, access-restricted, and retained only for the minimum period necessary during the audit window. User deletion requests received during this window should be acknowledged immediately. Where deletion is legally or operationally deferred, the system should provide a plain-language explanation of the reason for deferral, execute deletion at the close of the required retention window where permissible, and provide confirmation to the user.

**Implementation notes**

Retention clocks should reset only on substantively new user interactions, not on background system actions, passive analytics, or automated maintenance processes. Users should have access to a dashboard summarizing retained content by tier, including (a) full content listings for Tier 1 and Tier 2, (b) privacy-preserving audit-status information for Tier 3 without direct re-display of crisis content, and (c) derived-feature listings with plain-language interpretation for Tier 4. Where feasible, the dashboard should support one-click deletion at the tier or item level. Dashboard design for Tier 3 should follow trauma-informed principles and avoid direct re-display of crisis content that may trigger re-exposure. Where regulatory frameworks impose stricter defaults, those defaults govern. Documented deviations from these illustrative windows should be disclosed through periodic transparency reporting.

**References**

1. Regulation (EU) 2016/679 of the European Parliament and of the Council of 27 April 2016 on the protection of natural persons with regard to the processing of personal data and on the free movement of such data (General Data Protection Regulation), Articles 5, 6, 15, and 17. Off J Eur Union. 2016;L119:1-88. <https://eur-lex.europa.eu/eli/reg/2016/679/oj> [accessed 2026-04-12]
2. US Department of Health and Human Services. HIPAA Privacy Rule: right of access to designated record sets and administrative documentation requirements, 45 CFR §§164.501, 164.524, and 164.530(j). <https://www.ecfr.gov/current/title-45/subtitle-A/subchapter-C/part-164> [accessed 2026-04-12]
3. National Institute of Standards and Technology. NIST Privacy Framework: A Tool for Improving Privacy through Enterprise Risk Management, Version 1.0. Gaithersburg, MD: US Department of Commerce; 2020. doi:10.6028/NIST.CSWP.01162020
