# Supplementary material for: Ethical Considerations in Personal Health Large Language Models
Source: J Med Internet Res. 2026 Jun 17;28:e92240. doi: 10.2196/92240 (PMC13324317; doi:10.2196/92240)
Supplement: Multimedia Appendix 6 [file jmir_v28i1e92240_app6.docx]

**Multimedia Appendix 6.**

**Illustrative Scope Boundaries: Permitted Versus Clinician-Dependent Functions**

This appendix specifies illustrative scope boundaries for PH-LLM functions, presented in two complementary tables. Table 6-1 provides a conceptual categorization of PH-LLM support functions versus those requiring human professional agency, with the clinical and ethical rationale for professional dependence. Table 6-2 provides operational specifications for each function category, including permitted PH-LLM behaviors, behaviors that should be redirected or partitioned, and illustrative prompts. Function category boundaries are illustrative; deployers should refine boundaries based on the deployment context, WHO guidance on the governance of artificial intelligence for health [1], applicable medical device frameworks (see Multimedia Appendix 3), and the risk-tier classification developed in the main text.

Table 6-1. PH-LLM scope boundaries: informational support versus professional agency

| PH-LLM support functions (appropriate) | Human professional agency (requires clinician) | Clinical and ethical rationale |
| --- | --- | --- |
| Health literacy support and education | Diagnosis and differential assessment | Diagnosis requires physical examination, contextual synthesis, and accountability for diagnostic error |
| Symptom logging and general triage information | Prognosis and disease trajectory counseling | Prognostic judgments involve high uncertainty, value-sensitive communication, and risk of harm from misestimation |
| Medication reminders and adherence support | Prescribing, dose titration, and regimen optimization | Prescribing and dose changes are regulated clinical acts requiring licensure, monitoring, and legal responsibility |
| Wellness and lifestyle coaching | Interpretation of laboratory and imaging results | Interpretation requires clinical context, longitudinal comparison, and integration with examination and comorbidities |
| Care navigation and resource guidance | Crisis intervention and stabilization by trained human responders | Acute crises (eg, suicidality) require real-time human judgment, duty of care, and often legal authority to ensure safety |
| Option grid presentation for preference elicitation | High-stakes shared decision-making and consent | High-stakes choices require a therapeutic relationship, elicitation of values, and accountable deliberation about trade-offs |

Note: These columns represent distinct functional categories rather than direct substitution relationships. Boundary determinations may require dynamic adjustment based on user acuity, the evolving regulatory environment, and the availability of professional clinical resources or trained crisis-response services.

**Table 6-2.** Illustrative function categories with permitted versus redirected PH-LLM behaviors

| **Function category** | **PH-LLM may support** | **PH-LLM should redirect** | **Illustrative prompt** |
| --- | --- | --- | --- |
| Health information | Plain-language explanation of conditions, medications, and procedures based on curated authoritative sources; signposting to verified resources | Application of information to the user's specific case as diagnosis or treatment recommendation | "What is atrial fibrillation?" (support); "Could these symptoms mean I have atrial fibrillation?" (redirect) |
| Symptom navigation | Generic triage education; guidance on when to seek emergency, urgent, or routine care | Individual symptom interpretation as definitive triage decision | "When should chest pain be considered an emergency?" (support); "Is my chest pain an emergency?" (redirect with safety messaging) |
| Medication queries | General information about a medication's purpose, common side effects, and standard administration; reminder support | Dose adjustment, contraindication assessment for the user's regimen, recommendation to start, stop, or change a medication | "What is metformin used for?" (support); "Should I increase my metformin dose?" (redirect) |
| Mental health support | Validated coping-skill education, psychoeducation, mood tracking, sleep hygiene guidance | Psychiatric diagnosis, psychotherapy substitution, medication recommendations, or sustained crisis counseling beyond immediate handoff. | "What is a grounding exercise for anxiety?" (support); "Do I have major depression?" (redirect) |
| Lifestyle and self-management | Evidence-informed nutrition, physical activity, sleep, and behavior-change guidance | Individualized prescriptions for users with complex comorbidities or contraindications | "What does a balanced plate look like?" (support); "What should my diabetic renal-failure diet be?" (redirect) |
| Laboratory and imaging results | Plain-language explanation of what a test measures | Interpretation of an individual user's results | "What does HbA1c measure?" (support); "My HbA1c is 8.2%. What does this mean?" (redirect for clinical interpretation) |
| Preventive care | Evidence-based screening intervals from authoritative guidelines(for example, US Preventive Services Task Force [2]) or jurisdictionally relevant equivalents | Personalized screening decisions for users with risk-modifying conditions | "When are mammograms generally recommended?" (support); "Should I get screened earlier given my family history?" (redirect) |
| Crisis content | Immediate acknowledgment, regionally appropriate crisis resources [3], and handoff to human support.* | Sustained crisis counseling as a substitute for professional or emergency response | Any disclosure of self-harm, suicidality, or interpersonal violence triggers crisis protocol (Multimedia Appendix 7) |

*Crisis responses may include a brief safety check-in to assess acute risk, but should not become sustained counseling or a substitute for emergency or professional care.

**Multi-intent prompts**

When a single prompt combines a permitted and a redirected function, such as a sleep log submitted with a request to interpret a clinical questionnaire score, the system should use a partitioned response: provide the supported function, such as sleep coaching, while explicitly declining the redirected function, such as individualized questionnaire interpretation, and naming the appropriate professional resource. Blanket refusal of the entire prompt is discouraged.

**Function priority in mixed-intent interactions**

Where a single interaction implicates multiple function categories, the following priority order governs response generation: (1) crisis content always takes precedence over all other functions and triggers the crisis protocol regardless of other simultaneously raised topics; (2) safety-relevant medication or symptom queries take precedence over educational content; and (3) supported and redirected functions outside the crisis pathway can be addressed through a partitioned response within the same turn.

**Boundary cases**

Where a prompt occupies a genuine gray zone, such as “I have chest pain that comes and goes; should I be worried?”, the system should default to the more conservative redirect treatment and surface the rationale to the user, while continuing to provide whatever permitted information is helpful, such as a plain-language explanation of when chest pain is typically considered urgent. A conservative redirect approach should not preclude safe adjacent education; where appropriate, the system may still provide general information while directing the user to professional assessment.

**References**

1. World Health Organization. Ethics and governance of artificial intelligence for health: Guidance on large multi-modal models. Geneva: WHO; 2025. https://www.who.int/publications/i/item/9789240084759 [accessed 2026-04-12]
2. US Preventive Services Task Force. Recommendations. Rockville, MD: Agency for Healthcare Research and Quality. https://www.uspreventiveservicestaskforce.org/uspstf/recommendation-topics [accessed 2026-04-12]
3. Substance Abuse and Mental Health Services Administration. 988 Suicide & Crisis Lifeline. Rockville, MD: SAMHSA. https://988lifeline.org/ [accessed 2026-04-12]
