# Supplementary material for: Ethical Considerations in Personal Health Large Language Models
Source: J Med Internet Res. 2026 Jun 17;28:e92240. doi: 10.2196/92240 (PMC13324317; doi:10.2196/92240)
Supplement: Multimedia Appendix 7 [file jmir_v28i1e92240_app7.docx]

**Multimedia Appendix 7.**

**Adverse-Event Severity Categories and Response Pathways**

This appendix specifies illustrative severity categories for PH-LLM adverse events and associated graduated response pathways. Categories should be calibrated to deployment context; the framework below applies to consumer-facing direct-to-consumer PH-LLMs and is adapted from established medical-device risk-management and adverse-event terminology principles [1,2], usability-engineering considerations for safety-related use errors [3], and AI risk-management guidance [4].

**Table 7-1.** Adverse-event severity categories and graduated response pathways

| **Severity** | **Defining criteria** | **Illustrative examples** | **Internal response** | **External notification** | **Response timeline** |
| --- | --- | --- | --- | --- | --- |
| Critical | Imminent or actual serious harm; failure of a designated safety-critical function | Failure to detect explicit suicidal intent; harmful self-harm guidance; medication recommendation creating severe-harm potential, such as recommending a contraindicated drug combination, unsafe dose escalation, or delayed emergency care | Immediate crisis-response protocol; graduated suspension (session-level, feature-level within 24 hours, or temporary global if non-isolable) of implicated functionality; safety-team escalation | Regulator or oversight body within 72 hours where required; affected-user or public advisory within 7 days when legally and ethically appropriate, unless restricted by privacy obligations or active investigation | Root-cause analysis within 30 days; published summary within 60 days, redacted as needed for privacy or active investigation |
| Major | Significant safety-relevant failure without imminent harm | Hallucinated clinical claim; pharmacological misstatement; unauthorized disclosure of identifiable or sensitive health information without imminent harm; confirmed, safety-relevant, and reproducible fairness disparity (see Multimedia Appendix 1) | Targeted investigation; patch development; UI flagging or temporary functional limitation | Next quarterly transparency report; earlier notification where legally required or where user action is needed | Remediation within 30 days |
| Moderate | Function-level failure without direct safety implication | Assistive-technology accessibility failure; non-crisis localization error; intermittent low-risk guardrail bypass without unsafe clinical content | Documented investigation; standard maintenance or targeted usability fix | Annual transparency report, or earlier disclosure when the issue affects a vulnerable user group or requires user action | Remediation within 90 days |
| Minor | Non-safety operational issues | UI bugs; latency; non-clinical content errors | Standard product maintenance | Routine release notes | Standard product cycle |

**Cross-cutting principles**

Functionality suspension should be interpreted as a graduated response rather than default user lockout or automatic global shutdown. Depending on the scope of the safety failure, suspension may occur at three operational levels: session-level containment, which limits or terminates the affected conversation thread while preserving general system access; feature-level suspension, which disables the implicated functionality while maintaining unrelated safe functions; and temporary global suspension, which is reserved for system-wide critical safety risks that cannot be isolated to a specific thread or feature. User lockout without alternative crisis resources is inappropriate at any severity tier. Feature-level suspension is preferred over global service suspension where the implicated functionality can be isolated. Suspension decisions should consider the potential harm of removing access for users who depend on the service and should be paired with referral to alternative resources. Material, disputed, or recurrent severity classifications should be reviewable by an independent safety oversight body, with periodic sampling of lower-severity cases. Deployers operating without such a body should designate an alternative review pathway structurally separate from product and engineering leadership.

**Population-specific adjustments**

Adverse events involving minor users should receive heightened review. Major or Critical classification should apply when the event involves crisis-response failure, harmful-content delivery, unlawful disclosure of sensitive minor data, or other material safety, privacy, or safeguarding risks, consistent with Multimedia Appendix 4. Adverse events affecting users from groups with documented disparity identified through fairness monitoring (see Multimedia Appendix 1) should be reviewed for upward severity reclassification when patterns suggest systemic rather than individual failure.

**Reporting flow**

Reports may originate from users, caregivers, clinicians, third-party researchers, civil-society organizations, or internal monitoring. All reports should enter a unified triage queue, with severity assessment by trained safety reviewers according to the timelines specified above. Initial acknowledgment to the reporter should occur within 24 hours for Critical events, 7 days for Major events, and 14 days for Moderate events; acknowledgment is independent of, and precedes, investigation and remediation. Severity reclassification during investigation should be documented with rationale.

**References**

1. International Organization for Standardization. ISO 14971:2019 Medical devices — Application of risk management to medical devices. Geneva: International Organization for Standardization; 2019. https://www.iso.org/standard/72704.html

2. International Medical Device Regulators Forum. IMDRF terminologies for categorized adverse event reporting (AER): terms, terminology and codes. IMDRF/AE WG/N43FINAL:2020. Geneva: International Medical Device Regulators Forum; 2020. https://www.imdrf.org/documents/terminologies-categorized-adverse-event-reporting-aer-terms-terminology-and-codes

3. International Electrotechnical Commission. IEC 62366-1:2015 Medical devices — Part 1: Application of usability engineering to medical devices. Geneva: International Electrotechnical Commission; 2015. Amendment 1:2020. https://webstore.iec.ch/en/publication/59980

4. National Institute of Standards and Technology. Artificial Intelligence Risk Management Framework (AI RMF 1.0). Gaithersburg, MD: US Department of Commerce; 2023. doi:10.6028/NIST.AI.100-1. https://www.nist.gov/publications/artificial-intelligence-risk-management-framework-ai-rmf-10
