# Supplementary material for: Ethical Considerations in Personal Health Large Language Models
Source: J Med Internet Res. 2026 Jun 17;28:e92240. doi: 10.2196/92240 (PMC13324317; doi:10.2196/92240)
Supplement: Multimedia Appendix 8 [file jmir_v28i1e92240_app8.docx]

**Multimedia Appendix 8.**

**Feasibility, Cost Categories, and Implementation Trade-offs**

This appendix provides a qualitative feasibility framework for implementing the governance mechanisms proposed in the main text. Because published PH-LLM-specific compliance-cost evidence remains limited, cost implications are presented as qualitative categories rather than validated estimates. This appendix therefore does not assign precise monetary values. Instead, it clarifies relative implementation burden, institutional capacity requirements, scalability constraints, incentive alignment, and governance trade-offs across different deployer types [1-8].

**Approximate cost implications**

Near-term safeguards, such as role disclosure, scope labeling, basic safety signposting, and crisis-resource links, are generally implementable through interface or workflow changes. However, nonpersistent handling of sensitive interactions and basic adverse-event reporting may require additional privacy engineering, logging controls, and triage workflows. These measures are still likely to be less resource-intensive than independent certification, external audits, or continuous monitoring infrastructure [1,2].

By contrast, independent audits, structured red-team testing, certification preparation, continuous postdeployment monitoring, fairness audits, version-aware change control, and independent safety oversight are likely to impose moderate to high recurring costs. These requirements may be manageable for large technology platforms or well-resourced incumbents but may be more difficult for startups, academic deployers, small hospitals, and resource-constrained settings [3-6]. Proportionate certification, shared evaluation infrastructure, audit reciprocity, and reduced fixed-cost pathways may therefore be needed to avoid concentrating compliance capacity among large incumbents.

**Institutional capacity differences**

Implementation feasibility will vary substantially by deployer type. Large technology platforms may be able to absorb the costs of certification preparation, safety engineering, red-team testing, legal review, and continuous monitoring as part of broader compliance infrastructure. Mid-size or well-resourced startups may be able to implement core safeguards but may require external support for independent audits, fairness monitoring, and legal adaptation across jurisdictions. Small startups, academic deployers, hospitals, and nonprofit developers may lack dedicated safety engineering, regulatory affairs, and audit teams. Resource-constrained settings, including low- and middle-income health systems and small clinical institutions, may face additional barriers related to workforce shortages, limited technical infrastructure, and lack of accredited local oversight bodies.

Accordingly, a single uniform compliance pathway may unintentionally favor large incumbents and reduce access to beneficial PH-LLM functions in underserved settings. A proportionate approach should distinguish safeguards that can be implemented immediately from those requiring longer-term institutional infrastructure.

**Scalability constraints**

Governance mechanisms differ in scalability. Interface-level safeguards, such as nonhuman identity disclosure, scope labeling, and basic safety messaging, are relatively scalable because they can be embedded into product design. Standardized adverse-event reporting and transparency reporting are moderately scalable but require workflow support, triage capacity, and consistent severity classification. Continuous model monitoring, subgroup fairness audits, independent red-team testing, and external certification are less scalable because they require specialized expertise, recurring evaluation, and institutional coordination [1-4].

Scalability is especially challenging for PH-LLMs because system behavior may change after deployment through model updates, retrieval-source changes, prompt-template modifications, plugin integration, or interface redesign. Governance should therefore include version-aware monitoring and predefined triggers for reassessment, while avoiding overly burdensome reassessment requirements for minor changes that do not affect health-related functionality [3].

**Incentive alignment and regulatory realism**

The feasibility of PH-LLM governance depends not only on technical standards but also on incentives. Developers and deployers may have limited incentives to participate in certification if certification increases cost without affecting distribution, reimbursement, procurement, or liability exposure. For this reason, certification participation may need to be linked to market-access mechanisms, such as app-store distribution, platform hosting, payer or insurer reimbursement, institutional procurement, or eligibility for limited liability safe harbor [4-6].

However, regulatory realism requires acknowledging that not all jurisdictions have accredited auditors, dedicated AI regulators, or sufficient public funding to enforce comprehensive oversight. In such settings, interim approaches may include self-attestation against baseline standards, public model or system cards, shared evaluation consortia, regional audit hubs, regulatory sandboxes, and recognition of certification or audits performed in higher-capacity jurisdictions. These approaches should be understood as transitional mechanisms rather than substitutes for robust oversight [1,2,6].

**Risks of under-regulation and over-regulation**

The framework must balance two opposing risks. Under-regulation may allow safety-critical failures, privacy harms, biased recommendations, and crisis-management failures to propagate at scale, particularly among vulnerable users. It may also permit developers to market clinically relevant systems as general wellness tools to avoid scrutiny.

Over-regulation may also create harms. Excessively burdensome certification or audit requirements may reduce access, slow beneficial innovation, increase compliance costs, and consolidate the market around large firms with greater legal and technical resources. Smaller developers serving local, culturally specific, or underrepresented populations may be disproportionately affected. In addition, organizations may substitute documentation for substantive safety work if compliance becomes a checklist exercise rather than a learning-oriented governance process. These trade-offs support a risk-proportionate and capacity-sensitive implementation pathway, elaborated in the implementation principle below.

Table 8-1. Illustrative feasibility categories for PH-LLM governance mechanisms

| Governance mechanism | Examples | Relative cost burden | Capacity requirements | Implementation status |
| --- | --- | --- | --- | --- |
| Basic role and scope disclosure | Nonhuman identity disclosure; nonclinical role statement; scope labeling | Low | Interface design, legal review, health-literacy testing | Near-term feasible |
| Basic safety signposting | Crisis-resource links; emergency-care reminders; clinician-referral prompts | Low to moderate | Regional resource mapping, periodic updating, usability testing | Near-term feasible |
| Basic adverse-event reporting | In-app harm-reporting channel; user and clinician reports; severity triage | Low to moderate | Reporting workflow, triage staff, response-time commitments | Near-term feasible |
| Tiered consent and retention defaults | Sensitivity-tier classification; nonpersistent mode; deletion controls | Moderate | Privacy engineering, legal review, data-governance infrastructure | Near-term to mid-term feasible |
| Fairness monitoring | Subgroup audits; counterfactual vignette testing; disparity triggers | Moderate | Fairness expertise, subgroup data governance, privacy safeguards | Mid-term feasible |
| Red-team testing | Prompt-injection testing; crisis-bypass attempts; safety stress testing | Moderate to high | Safety engineering, clinical review, adversarial testing expertise | Mid-term feasible |
| Certification preparation | Documentation, model cards, evaluation reports, audit readiness | Moderate to high | Regulatory affairs, evaluation infrastructure, technical documentation | Mid-term feasible |
| Independent external audits | Third-party compliance review; safety and fairness audits | High recurring | Accredited auditors, funding model, audit standards | Longer-term or high-tier requirement |
| Continuous postdeployment monitoring | Drift monitoring; adverse-event trend analysis; version-aware change control | High recurring | Monitoring infrastructure, analytics capacity, governance staff | Longer-term or high-tier requirement |
| Independent safety oversight | Safety advisory board; independent review of critical events | High recurring | Independence-preserving funding, governance authority, conflict-of-interest controls | Longer-term or high-tier requirement |

**Near-term feasible actions versus longer-term aspirational governance**

Near-term feasible actions include nonhuman identity disclosure, role and scope labeling, crisis-resource signposting, basic adverse-event reporting, exclusion of Tier 3 crisis-related content from model training or fine-tuning datasets, and user-facing data controls. These safeguards are relatively implementable because they can be introduced through interface design, workflow changes, and baseline privacy governance.

Longer-term aspirational governance elements include harmonized certification reciprocity, accredited cross-jurisdictional audit infrastructure, sustainably funded independent safety advisory boards, population-level longitudinal outcomes monitoring, and enforceable market-access conditionality. These mechanisms require broader institutional coordination, stable funding, and legal or regulatory authority. They should therefore be framed as conceptual or normative governance proposals rather than immediate universal requirements.

**Implementation principle**

Implementation should follow a proportionate pathway informed by risk-management and trustworthy-AI governance principles [7,8]: baseline safeguards should be treated as minimum expectations across PH-LLM deployments, while higher-risk systems should face more demanding certification, auditability, postdeployment monitoring, and independent oversight requirements. This approach aims to preserve user safety and accountability without imposing uniform burdens that could reduce access, suppress smaller innovators, or divert resources from substantive safety work to formal compliance alone.

**References**

1. National Institute of Standards and Technology. Artificial Intelligence Risk Management Framework (AI RMF 1.0). Gaithersburg, MD: US Department of Commerce; 2023. doi:10.6028/NIST.AI.100-1[accessed 2026-04-10]
2. World Health Organization. Regulatory considerations on artificial intelligence for health. Geneva, Switzerland: World Health Organization; 2023. https://www.who.int/publications/i/item/9789240078871 [accessed 2026-04-10]
3. US Food and Drug Administration. Marketing submission recommendations for a predetermined change control plan for artificial intelligence-enabled device software functions: guidance for industry and Food and Drug Administration staff. Silver Spring, MD: US Food and Drug Administration; 2025. https://www.fda.gov/regulatory-information/search-fda-guidance-documents/marketing-submission-recommendations-predetermined-change-control-plan-artificial-intelligence [accessed 2026-04-10]
4. Coalition for Health AI. Responsible AI Guide (RAIG). Boston, MA: Coalition for Health AI; 2024. https://www.chai.org/workgroup/responsible-ai/responsible-ai-guide-raig-and-raig-executive-summary[accessed 2026-04-10]
5. National Telecommunications and Information Administration. Artificial Intelligence Accountability Policy Report. Washington, DC: US Department of Commerce; 2024. https://www.ntia.gov/issues/artificial-intelligence/ai-accountability-policy-report [accessed 2026-04-10]
6. European Parliament and Council of the European Union. Regulation (EU) 2024/1689 of the European Parliament and of the Council of 13 June 2024 laying down harmonised rules on artificial intelligence. Off J Eur Union. 2024;L 2024/1689. https://eur-lex.europa.eu/eli/reg/2024/1689/oj [accessed 2026-04-10]
7. International Organization for Standardization. ISO 14971:2019 Medical devices — Application of risk management to medical devices. Geneva, Switzerland: International Organization for Standardization; 2019. https://www.iso.org/standard/72704.html
8. Organisation for Economic Co-operation and Development. Recommendation of the Council on Artificial Intelligence. OECD/LEGAL/0449. Paris, France: Organisation for Economic Co-operation and Development; 2019, amended 2024. https://legalinstruments.oecd.org/en/instruments/OECD-LEGAL-0449 [accessed 2026-04-10]
